# CMA‐mediated USP9X degradation promotes SHH medulloblastoma progression by facilitating SUFU ubiquitination

**DOI:** 10.1002/ctm2.70635

**Published:** 2026-03-08

**Authors:** Binbin Gao, Qin Zhu, Lun Kuang, Jiahui Li, Qingyue Meng, Bo'ang Han, Yu Wang, Xinyi Zhang, Xiangxiang Zhang, Xinfa Wang, Tingting Yu, Shen Yue, Chen Liu

**Affiliations:** ^1^ Department of Medical Genetics Nanjing Medical University Nanjing China; ^2^ Department of Neurosurgery Children's Hospital of Nanjing Medical University Nanjing China

**Keywords:** medulloblastoma, SHH, SUFU, USP9X

## Abstract

**Background:**

Medulloblastoma (MB) represents the most prevalent malignant paediatric brain tumour, characterised by the sonic Hedgehog molecular subtype (SHH‐MB), which is driven by aberrant activation of the SHH signalling cascade. Suppressor of fused (SUFU), a core member of SHH signal transduction, functions as a tumour suppressor by inhibiting the activity of transcription factors glioma‐associated oncogene homologue (GLI)‐triggered SHH signalling pathway. While ubiquitin‐mediated proteasomal degradation of SUFU has been shown to modulate SHH signalling, the regulatory factors involved in SUFU deubiquitination and their specific roles in MB pathogenesis remain largely undefined.

**Methods:**

Mass spectrometry and co‐immunoprecipitation were employed to identify the interaction between USP9X and SUFU. Clinical correlation analyses were conducted using MB tissue microarrays and publicly available datasets. Ubiquitination assays, functional cell‐based experiments, and orthotopic xenograft models were performed to evaluate the biological role of USP9X in SHH‐MB. Chaperone‐mediated autophagy (CMA) inhibitors were utilised to investigate their regulatory effects on USP9X expression and SHH‐MB progression.

**Results:**

Mass spectrometry identified the deubiquitinase ubiquitin‐specific protease 9X (USP9X) as a previously unrecognised SUFU‐binding partner. Notably, USP9X and SUFU exhibit a positive correlation in a MB tissue array, with both exhibiting low expression levels that are associated with adverse prognostic outcomes. Loss of USP9X in SHH‐MB enhances cell proliferation in vitro as well as orthotopic MB xenograft tumourgenicity in vivo. Mechanically, USP9X deubiquitinates and stabilises SUFU, thereby negatively regulating SHH signal transduction. Interestingly, SHH signalling promotes SUFU ubiquitination through CMA‐dependent degradation of USP9X protein levels, facilitating pathway activation. Combined inhibition of CMA and SHH pathway had a synergically therapeutic effect on SHH‐MB.

**Conclusions:**

These results establish CMA and USP9X as pivotal regulators of SHH medulloblastoma MB progression, emphasising their potential as novel therapeutic targets for medulloblastoma, and combinatorial inhibition of CMA and Smoothened (SMO) may provide a strategy to overcome intrinsic or acquired resistance to SMO monotherapy in SHH‐MB.

**Key points:**

- Deubiquitinase USP9X suppresses SHH Medulloblastoma progression by facilitating SUFU ubiquitination.
- SHH promotes USP9X degradation through the CMA pathway.
- Combined inhibition of CMA and SHH pathway had a synergically therapeutic effect on SHH Medulloblastoma.

## Graphical abstract

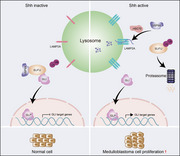

## 1 INTRODUCTION

Medulloblastoma (MB) represents the most prevalent malignant paediatric brain tumour and is molecularly categorised into four principal subgroups based on gene expression profiling and deep sequencing analysis: wingless‐related integration site, sonic Hedgehog (SHH), group 3 and group 4.^1^, ^2^, ^3^, ^4^ The SHH molecular subtype (SHH‐MB), which accounts for around 30% of all cases, is characterised by constitutive activation of the SHH signalling pathway.^5^, ^6^ Within this signalling cascade, binding of the SHH ligand to its receptor Patched 1 (PTCH1) alleviates repression of the transmembrane protein Smoothened (SMO), triggering downstream signalling events.^7^, ^8^, ^9^ Activated SMO facilitates the release of glioma‐associated oncogene homologue (GLI) transcription factors from suppressor of fused (SUFU)‐mediated inhibition. Subsequently, GLI proteins enter the nucleus as full‐length activators, where promote transcription of SHH target genes, including *GLI1* and *PTCH1*.^10^

SUFU, as a key downstream core member of the SHH signalling pathway, inhibits the activity of transcription factors GLI (including GLI1, GLI2 and GLI3)‐mediated SHH signalling pathway, thereby participating in mammalian embryonic development and tumour progression.^11^, ^12^, ^13^, ^14^ Several reports have demonstrated that *Sufu* knockout mice died at E9.5 due to neural tube defects associated with excessive SHH pathway activation.^15^, ^16^ Moreover, *Sufu* heterozygous mice, in a background of Tp53 deficiency, spontaneously develop MB as well as rhabdomyosarcoma.^17^ In humans, loss of *SUFU* has been implicated in cancer initiation and progression, including basal cell carcinoma, meningioma and MB.^18^, ^19^, ^20^ Notably, it has been reported that over 50% of desmoplastic or nodular MB patients under the age of 3 years were due to germline SUFU mutations.^21^ Accumulating evidence demonstrates that tumour suppressor SUFU may be an ideal target for treating SHH‐MB.

As one of the most extensive post‐translational modifications, protein modification by ubiquitin is a dynamic and reversible processes that regulate protein stability and function in cancer.^22^ SHH signalling promotes SUFU ubiquitination and subsequently degradation through the ubiquitin‒proteasome pathway.^23^ And several studies have demonstrated that the E3 ligases FBXL17, ITCH, ROC1 and LNX1 have been identified as modifiers of SUFU ubiquitination.^24^, ^25^, ^26^, ^27^ These E3 ligases promote SUFU ubiquitination, which in turn affects SUFU protein stability or its ability to bind to GLI proteins, thereby modulating SHH pathway activity across various tumour types. However, to date, the deubiquitination mechanism of SUFU remains elusive.

In this study, we identified ubiquitin‐specific protease 9X (USP9X) interacts with SUFU through mass spectrometry analysis. USP9X belongs to the ubiquitin‐specific protease (USP) family and regulates the stability of target proteins by removing their ubiquitin moieties.^28^ It has been established as an oncogene or tumour suppressor in various cancers but has not been reported in MB.^28^ Here, we performed immunohistochemical analysis on a MB tissue microarray, revealing a significant positive correlation between USP9X and SUFU protein abundance. Further analysis of GEO datasets indicated that, among 763 patients, elevated USP9X and SUFU expression are associated with enhanced survival outcomes in SHH‐MB. Subsequent cellular functional assays and orthotopic xenograft experiments in murine models provided preliminary evidence that USP9X can inhibit the progression of SHH‐MB. Mechanically, the results shown that reducing USP9X expression led to a decrease in SUFU protein levels, reduced stability, increased binding affinity with ubiquitin molecules and further activation of SHH signalling. Interestingly, in SHH‐activated cells, USP9X protein levels were downregulated. Ultimately, we demonstrated that the reduction of USP9X mediated by SHH occurs via an alternative degradation mechanism, specifically chaperone‐mediated autophagy (CMA). These data support a model in which SHH, subsequent to the CMA‐mediated degradation of USP9X, enhances the ubiquitin‐mediated degradation of SUFU, thereby activating the SHH signalling pathway. Importantly, combined inhibition of CMA and SHH signalling produced synergistic therapeutic effects in SHH‐MB models, highlighting the CMA–USP9X regulatory axis as a potential therapeutic vulnerability.

## 2 MATERIALS AND METHODS

### 2.1 Cells, plasmids and siRNAs

Wild‐type (WT) and Gli1‐deficient mouse embryonic fibroblasts (MEFs) were kindly provided by the Wade Bushman laboratory. Ptch1^−/−^ and Sufu^−/−^ MEFs were obtained from the Rune Toftgard Laboratory. HEK293, NIH3T3, DAOY and ONS‐76 cell lines were acquired from ATCC. Full‐length and mutant constructs of Usp9x (Myc‐Usp9x^WT^, Myc‐Usp9x^C1566A^, Myc‐Usp9xN1, Myc‐Usp9xN2, Myc‐Usp9xC1, Myc‐Usp9xC2) were amplified by PCR and inserted into the pRK5 vector (RRID: Addgene 32693). pReceiver‐M14‐Lamp2a and pReceiver‐M14‐Hsc70 vectors were procured from Jinbeijin Biotechnology. siRNAs targeting SUFU, USP9X and LAMP2A were synthesised by GenePharma. The corresponding sequences are provided in Table S1.

### 2.2 Immunoblotting and cycloheximide chase assay

Cells were lysed on ice in RIPA buffer (150 mM NaCl, 50 mM Tris‒HCl [pH 7.5], 1 mM EDTA [pH 8.0], .5% sodium deoxycholate, 1% NP‐40, .1% SDS, 2% sodium fluoride, .5% sodium orthovanadate and protease inhibitor cocktail) for 30 min. Lysates were cleared by centrifugation at 14 000 × *g* for 20 min at 4°C. Protein concentrations were quantified using a bicinchoninic acid assay. Equal amounts of protein were denatured in SDS loading buffer, resolved by 8% sodium dodecyl sulfate‐polyacrylamide gel electrophoresis (SDS–PAGE), and electrotransferred onto polyvinylidene difluoride membranes. Membranes were blocked with 5% skim milk in tris‐buffered saline with tween 20 and incubated with primary antibodies overnight at 4°C, followed by horseradish peroxidase (HRP)‐conjugated secondary antibodies. Signal detection was performed using Clarity Western ECL substrate (Bio‐Rad), and band intensities were quantified using ImageJ software. The antibodies utilised were detailed in Table S2. To assess protein turnover, cells transfected with the specified plasmids were treated with cycloheximide (CHX) at a concentration of 10 µM (Sigma) to inhibit protein synthesis. Cells were harvested at indicated time points and analysed by immunoblotting.

### 2.3 Coimmunoprecipitation

Cells were lysed on ice in RIPA buffer for 30 min, followed by high‐speed centrifugation. Protein concentrations of the lysates were quantified, and subsequent immunoprecipitation (IP) was performed with anti‐FLAG M2 affinity gel (Sigma) or anti‐Myc antibody (Thermo Fisher) conjugated to Dynabeads Protein G (Thermo Fisher). Following extensive washing to remove nonspecific interactions, immunocomplexes were eluted and subjected to SDS–PAGE and immunoblot analysis.

### 2.4 Reverse transcription and real‑time PCR

Total RNA was extracted using RNAiso Plus reagent (TaKaRa) according to the manufacturer's instructions. Complementary DNA (cDNA) was synthesised using HiScript II Q RT Super Mix (Vazyme). Quantitative real‐time PCR (qPCR) was carried out using AceQ SYBR Green Master Mix (Vazyme) on a real‐time PCR system. Each reaction was performed in triplicate, and experiments were independently repeated three times. Primer sequences are provided in Table S3.

### 2.5 Mass spectrometry

Endogenous Sufu in MEF cells was immunoprecipitated using an anti‐Sufu antibody. Immunoprecipitates were validated by immunoblotting and subsequently subjected to gel‐free mass spectrometry analysis at the Nanjing Medical University Analytical Center to identify interacting proteins.

### 2.6 Immunohistochemistry

MB tissue microarrays were obtained from Nanjing Drum Tower Hospital. Antigen retrieval was achieved by heating sections in 10 mM sodium citrate buffer (pH 6.0). Sections were incubated with primary antibodies, followed by HRP‐conjugated secondary antibodies. Staining intensity and the proportion of positively stained cells were independently evaluated. A semiquantitative scoring system was applied: percentage scores (0–4) were multiplied by staining intensity to generate an overall immunohistochemistry score. Ki67 staining was performed to assess proliferative index in xenograft tumour tissues using a rabbit anti‐Ki67 antibody (Abcam).

### 2.7 Cell counting kit‑8, EdU and colony formation assays

For proliferation analysis, DAOY and ONS‐76 cells were seeded into 96‐well plates and cultured under standard conditions. Cell viability was measured at indicated time points using the Cell counting kit‑8 (CCK‐8) assay (Vazyme), and absorbance at 450 nm was recorded using a microplate reader. Experiments were conducted in quadruplicate and repeated three times.

In the 5‐ethynyl‐2'‐deoxyuridine (EdU) incorporation assays, cells were plated in 24‐well plates. EdU incorporation was detected using the Cell‐Light EdU kit (Ribo Bio) to assess cellular proliferation. Fluorescence microscopy was employed to capture images, and ImageJ software was utilised to quantify EdU‐positive cells across five distinct fields of view.

For clonogenic assays, DAOY and ONS‐76 cells were plated at densities of 800 cells per P60 dish and 1200 cells per well in six‐well plates, respectively, and allowed to grow for approximately 10 days. Colonies were fixed in paraformaldehyde, stained with crystal violet, and counted manually.

### 2.8 Tumoursphere assays

A total of 5000 cells were cultured in serum‐free neural stem cell medium supplemented with growth factors (epidermal growth factor and fibroblast growth factor) and B27 supplement. After 7 days, spheres exceeding 40 µm in diameter were counted under a microscope and defined as tumourspheres.

### 2.9 CRISPR‑Cas9 genome editing

Gene knockout was performed using CRISPR–Cas9 technology. Single‐guide RNAs (sgRNAs) were designed using an online CRISPR design platform. Ribonucleoprotein complexes were assembled using synthetic crRNA, tracrRNA, Cas9 nuclease and electroporation enhancer (IDT). The complexes were introduced into hiPSCs via nucleofection (Lonza). After recovery, single‐cell clones were expanded and screened by PCR, followed by Sanger sequencing to confirm successful gene editing. Primer and sgRNA sequences are listed in Table S4.

### 2.10 Establishment and treatment of the in vivo intracranial xenograft model

Orthotopic xenografts were established in 6‐week‐old non‐obese diabetic severe combined immunodeficient mice under anesthesia. A burr hole was drilled above the right cerebellar hemisphere, and luciferase‐labelled DAOY cells were stereotactically injected into the cerebellum. One week after implantation, bioluminescence imaging was performed using the in vivo imaging system (IVIS) Spectrum system (PerkinElmer) to confirm successful tumour establishment. Mice with confirmed intracranial tumour growth were randomly assigned to experimental groups. Control mice received intraperitoneal (i.p.) injections of vehicle solution containing 5% dimethyl sulfoxide, 30% polyethylene glycol, 5% Tween‐80 and 60% normal saline. In the treatment groups (three mice per group), GDC‐0449 (35 mg/kg) was administered via i.p. injection each morning, followed 4 h later by i.p. administration of VER155008 (4.37 mg/kg) and all‐trans retinoic acid (ATRA; 15 mg/kg). Treatments were administered once daily for 4 consecutive weeks. Body weight was recorded weekly, and drug dosages were adjusted accordingly based on the measured body weight. Tumour progression was evaluated by weekly bioluminescence imaging.

### 2.11 GST pull‐down assay

The glutathione s‐transferase (GST)‐tagged Sufu and HSC70 expression vector pGEX‐2T was successfully constructed and expressed in *Escherichia coli* BL21. Both the control GST and GST fusion proteins were induced using 1 mM IPTG (final concentration). The fusion proteins were subsequently incubated with BeyoGold GST‐tag purification resin for 1 h. Following a series of wash steps, the resin was incubated with 1 mL of cell lysate at 4°C for 2 h. The bound proteins were eluted and analysed by immunoblotting.

### 2.12 Analysis of publicly available datasets

Transcriptomic data from the human MB cohort GSE85217 were retrieved and analysed to evaluate associations with overall survival. Survival curves were constructed using the R2 platform (Kaplan–Meier module), with cutoff values determined automatically by the algorithm to achieve optimal group separation. Hazard ratios together with 95% confidence intervals were calculated, and statistical significance was assessed using the log‐rank test. Differences were considered statistically significant when *p*‐values were below. 05.

### 2.13 Statistics

Data were analysed using GraphPad Prism 9. Results are presented as mean ± SD. Statistical comparisons were performed using unpaired two‐tailed Student's *t*‐test or one‐way/two‐way analysis of variance where appropriate. A *p*‐value < .05 was considered statistically significant.

## 3 RESULTS

### 3.1 Deubiquitinase USP9X interacts with SUFU and exhibit a positive correlation in MB

**FIGURE 1:**
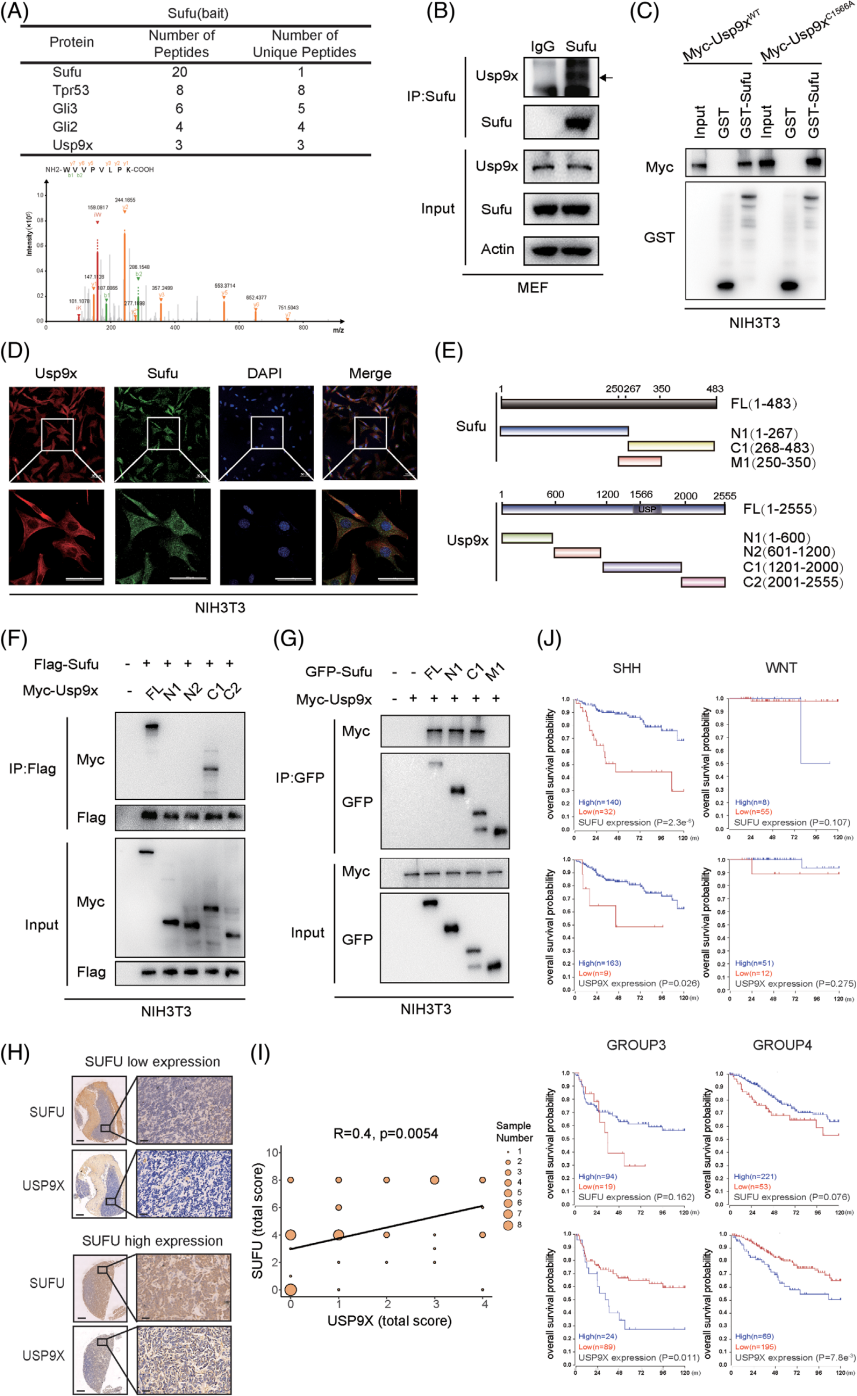
Usp9x binds to Sufu and is associated with favourable prognosis in medulloblastoma (MB). (A) Mass spectrometry analysis conducted in mouse embryonic fibroblast (MEF) cells revealed a list of proteins that interact with Sufu. Peptide identification was validated by matching experimental MS/MS spectra to theoretical b and y fragment ion series, and a representative spectrum of USP9X is shown. (B) Cell lysates obtained from MEF cells were subjected to immunoprecipitation (IP) using an antibody targeting Sufu, with immunoglobulin G (IgG) serving as the isotype control. (C) Purified Myc‐tagged Usp9x^WT^ and Usp9x^C1566A^ were incubated with either GST or GST‒Sufu attached to glutathione‐Sepharose beads. Proteins bound to the Sepharose were then analysed via immunoblotting using indicated antibodies. (D) Immunofluorescence showed the co‐localisation of Sufu and Usp9x in the cytoplasm. (E) Schematic representation of full‐length (FL) Usp9x, Sufu and their generated deletion mutants. (F) Flag‐Sufu and Myc‐tagged FL USP9X or its deletion mutants were cotransfected into NIH3T3 cells, and their interactions were examined through IP‐Western blotting. (G) Myc‐Usp9x and GFP‐tagged FL Sufu or its deletion mutants were cotransfected into NIH3T3 cells, and their interactions were examined through IP‐Western blotting. (H and I) Immunohistochemical analysis of protein expression levels of SUFU and USP9X in a MB tissue array. (J) Kaplan‒Meier plot illustrates the survival comparison between MB patients with low and high expression levels of USP9X.

To uncover regulators involved in Sufu ubiquitination, we performed mass spectrometry‐based interactome analysis in MEF and NIH3T3 cells. In MEF cells, the results not only confirmed the presence of known Sufu‐binding proteins like Gli2 and Gli3 but also unveiled the presence of a deubiquitinase known as Usp9x (Figure 1A). Similar outcomes were observed in NIH3T3 cells, indicating a consistent interaction between Usp9x and Sufu (data not shown). To validate these findings, IP experiments in MEF cells further affirmed the binding between endogenous Sufu and Usp9x (Figure 1B). Moreover, in our in vitro GST pull‐down assay, Usp9x displayed a distinct binding affinity for purified GST‒Sufu protein compared to GST alone, providing further evidence of the direct interaction between Sufu and Usp9x (Figure 1C, left panel). Additionally, immunofluorescence analysis demonstrated that while Sufu localised to both nucleus and cytoplasm, Usp9x was predominantly cytoplasmic, and substantial co‐localisation was observed in the cytoplasmic compartment (Figure 1D). To pinpoint the regions responsible for the Usp9x‐Sufu interaction, we generated various truncation mutants of Myc‐Usp9x and Sufu‐GFP (Figure 1E). The experimental outcomes highlighted that Usp9x interacts with Sufu primarily through its C1 domain sequence (amino acids 1200–2000), while Sufu binds to Usp9x through its N1‐terminal sequence (amino acids 1–267) and C1‐terminal region (amino acids 268–483) (Figure 1F,G). These collective results solidify the notion that the deubiquitinase Usp9x can indeed form a binding partnership with Sufu, presenting an avenue for exploring Sufu's deubiquitination processes.

Given the critical role of SUFU in the development of MB, we examined USP9X and SUFU protein expression in a MB tissue microarray (*n* = 48). A strong positive correlation between USP9X and SUFU levels was observed (Figure 1H,I). survival analysis of 763 MB patients from the GSE85217 dataset demonstrated that elevated USP9X expression predicts improved overall survival specifically in SHH‐subtype MB, mirroring the prognostic pattern of SUFU (Figure 1J). These findings suggest a clinically relevant USP9X–SUFU axis in SHH‐MB.

### 3.2 USP9X‒SUFU axis suppresses MB cell proliferation

To determine the functional relevance of this interaction, we transfected SHH‐MB DAOY cells with siSUFU, Myc‐Usp9x or both, and subsequently assessed their effect on cell proliferation (Figure S1A). The results revealed that, across various assays, including CCK‐8, EDU incorporation and colony formation assays, silencing SUFU led to an increase in MB cell proliferation, which is consistent with findings reported in other studies, while overexpression of Usp9x suppressed cell proliferation. Notably, when siSUFU and Myc‐Usp9x were co‐expressed, loss of SUFU can effectively reverse the suppression of cell proliferation associated with USP9X overexpression (Figure S1B‒F). To further validate these results, Flag‐SUFU, siUSP9X or both were transfected into DAOY and ONS‐76 cells and cell proliferation was re‐evaluated (Figures S2A and S3A). Consistently, overexpressing SUFU suppressed MB cell proliferation, while the overexpression of SUFU mitigated the increased proliferation induced by *siUSP9X* (Figures S2B‒D and S3B‒F).

**FIGURE 2:**
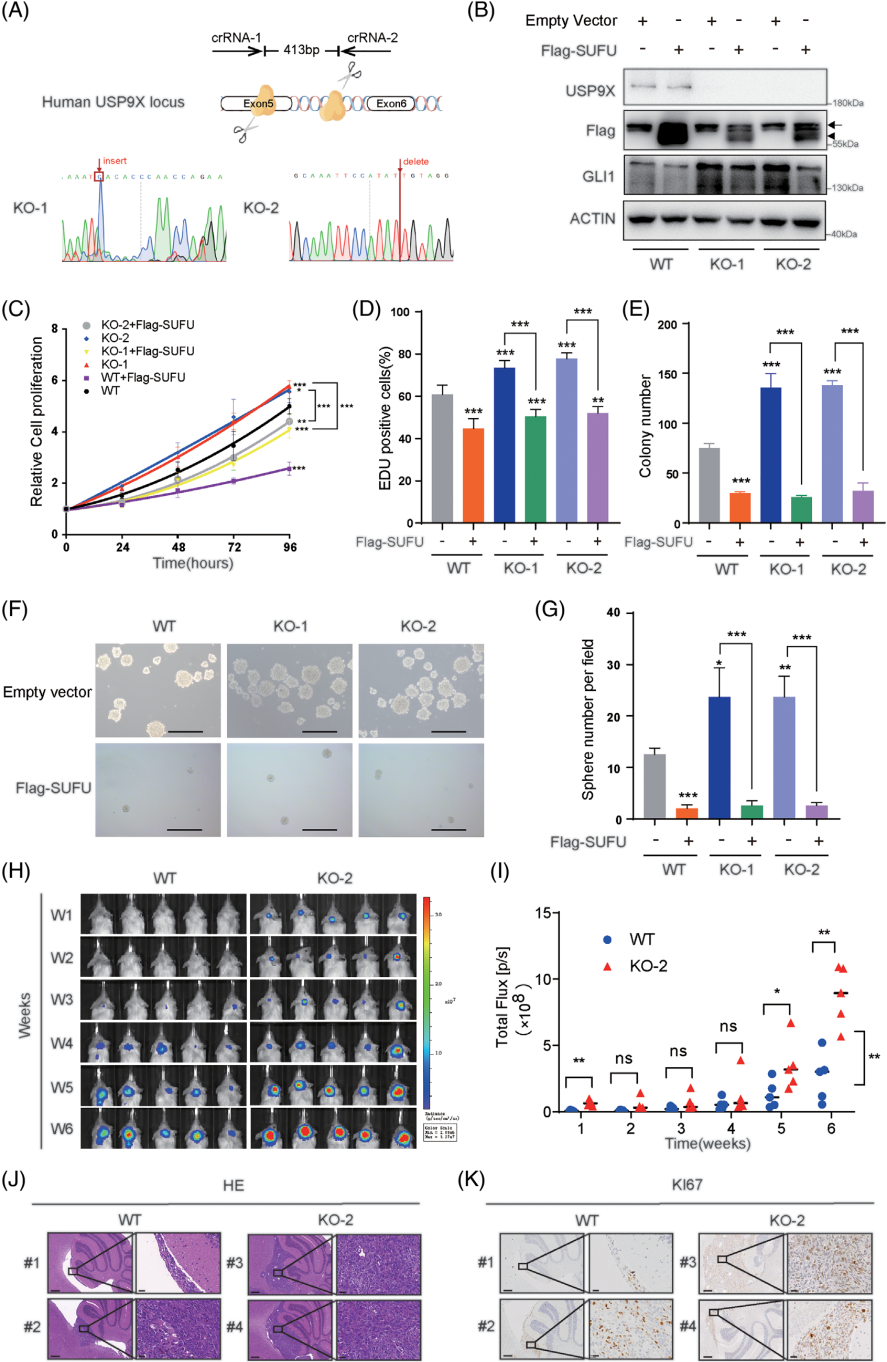
Loss of *USP9X* promotes medulloblastoma cell proliferation by downregulating SUFU. (A) Schematic diagram of CRISPR‐Cas9 gene editing technology knocking out *USP9X* in DAOY cells (upper). CRISPR‐edited *USP9X* gene shows altered nucleotide peaks in Sanger sequencing (lower). (B) Western blot validation of SUFU overexpression in *USP9X* knockout cells. Cell counting kit‑8 (CCK‐8) (C), EdU (D) and colony formation assay (E) were used to detect the effect of Flag‐SUFU overexpression on DAOY cell viability in *USP9X* knockout cells. (F and G) Sphere formation assay to detect the stemness of DAOY cells and statistical results. (H and I) Imaging and statistical analyses of orthotopic tumours derived from wild‐type (WT) and KO‐2 cells revealed that *USP9X* knockout cells exhibited enhanced proliferation ability. (J) Haematoxylin and eosin (HE) staining was performed to assess tumour formation in the cerebella of mice bearing WT and KO‐2 cells. (K) Immunohistochemical analysis was conducted to evaluate the levels of Ki67 in tumour tissues. ^*^ *p* < .05; ^**^ *p* < .01; ^***^ *p* < .001; ns, not significant.

To eliminate potential siRNA off‐target effects, CRISPR/Cas9‐mediated *USP9X* knockout DAOY cell lines were generated (KO‐1 and KO‐2) (Figure 2A,B). Cell proliferation assays conducted on WT, KO‐1 and KO‐2 cells after SUFU expression revealed that *USP9X* knockout cells (blue and red lines) exhibited significantly enhanced proliferation compared to WT cells (black line). Notably, reintroducing SUFU into the knockout cells resulted in a marked reduction in proliferation (grey and yellow lines) (Figure 2C). Consistent findings were observed in EDU (Figures 2D and S4A) and colony formation assays (Figures 2E and S4B). Furthermore, sphere‐forming assays demonstrated that USP9X‐deficient MB cells generated more tumour spheres than WT cells, whereas overexpression of Flag‐SUFU in DAOY cells significantly impaired their sphere‐forming capacity (Figures 2F,G and S3G,H). These results indicate that USP9X downregulation in MB cells enhances stemness and self‐renewal, with Flag‐SUFU overexpression reversing this effect. Overall, our findings confirm the successful generation of *USP9X* knockout DAOY cells and highlight that USP9X‒SUFU axis inhibits the proliferation of MB cells. To further elucidate the role of USP9X in the SHH‐MB in vivo, we established a mouse orthotopic tumour model by infecting WT and KO‐2 cells with a luciferase‐expressing virus, followed by implantation into the cerebella of SCID mice. The results indicated that *USP9X* knockout cells exhibited a significantly enhanced tumourigenic capacity compared to WT cells in the immunodeficient mice (Figure 2H‒J). Additionally, immunohistochemical analysis revealed an increased number of Ki67‐positive cells within the USP9X‐deficient MB tumour tissues (Figure 2K). These results demonstrate that USP9X functions as a tumour suppressor in SHH‐MB.

### 3.3 USP9X counteracts SUFU ubiquitylation

**FIGURE 3:**
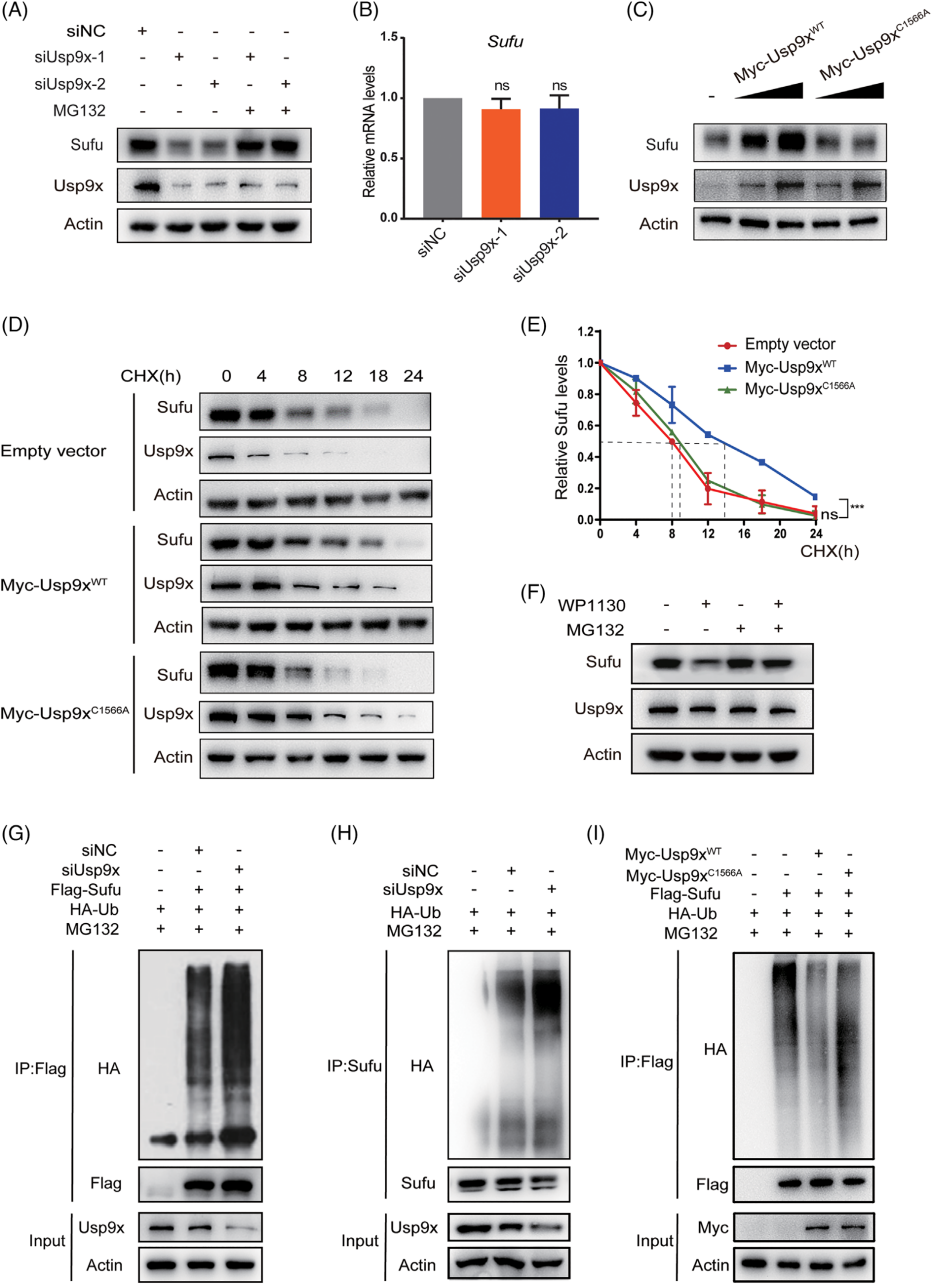
Usp9x deubiquitinates Sufu. (A) NIH3T3 cells transfected with the indicated siRNA were treated with or without MG132, and then Sufu and Usp9x were analysed. (B) NIH3T3 cells expressing siNC, siUsp9x‐1, siUsp9x‐2 were analysed by real‐time PCR. (C) Increasing amounts of Usp9x^WT^ and Usp9x^C1566A^ were transfected into NIH3T3 cells, followed by immunoblotting analysis. Western analysis (D) and quantification thereof (E) of Sufu protein levels in NIH3T3 cells transfected with Myc‐tagged Usp9x^WT^ or Usp9x^C1566A^ are shown. Protein synthesis was blocked with cycloheximide (CHX) treatment. (F) NIH3T3 cells were treated with or without WP1130 in the absence or presence of MG132, and cell lysates were subjected to immunoblotting analysis using indicated antibodies. (G) NIH3T3 cells were cotransfected with indicated siRNA, Flag‐Sufu and HA‐Ub in the presence of MG132, and cell lysates were subjected to immunoprecipitation (IP) with Flag beads followed by immunoblotting analysis. (H) NIH3T3 cells were cotransfected with indicated siRNA and HA‐Ub in the presence of MG132, and cell lysates were subjected to IP with Sufu antibody followed by immunoblotting analysis. (I) NIH3T3 cells were cotransfected with Flag‐Sufu, HA‐Ub and Myc‐tagged Usp9x^WT^ or Usp9x^C1566A^ in the presence of MG132, and cell lysates were subjected to IP with Flag beads followed by immunoblotting analysis. ^***^ *p* < .001; ns, not significant.

Usp9x is identified as a deubiquitinating (DUB) enzyme, which means it enhances the stability of specific proteins by removing ubiquitin molecules bound to them. Therefore, we designed two siRNAs targeting Usp9x and examined whether downregulating Usp9x in NIH3T3 cells would alter the protein levels of Sufu. The results showed a marked reduction of Sufu protein levels in both Usp9x knockdown groups, with mRNA levels remaining unaltered. Subsequently, siUsp9x‐1 was chosen for use in subsequent experiments. Moreover, this effect was almost completely rescued by the proteasome inhibitor MG132, indicating that Sufu reduction following Usp9x knockdown is primarily mediated by proteasomal degradation (Figure 3A,B). To validate these findings, we performed a gradient overexpression of Usp9x and found that Sufu protein levels increased consistently (Figure 3C). Furthermore, after inhibiting de novo protein synthesis using CHX, we assessed whether Usp9x affects the protein stability of Sufu. The results indicated that transfection of Usp9x slowed down the turnover rate of Sufu protein, making it more stable (Figure 3D,E).

The regulation of target protein stability by deubiquitinases depends on their DUB activity. Therefore, we used a small molecule inhibitor of Usp9x, WP1130, to inhibit its DUB activity and then measured Sufu protein levels. When Usp9x's DUB activity was inhibited, Sufu protein levels significantly decreased, while Usp9x protein levels remained unchanged. Again, MG132 could reverse this effect, underscoring the dependence of Sufu stability on Usp9x's deubiquitinase activity (Figure 3F). To exclude potential off‐target effects of the small‐molecule inhibitor, we constructed a Usp9x mutant vector (C1566A)^29^, ^30^, ^31^ with impaired deubiquitinase activity. Despite this mutant could still bind to Sufu (Figure 1C), it was unable to regulate Sufu protein levels and stability (Figure 3C,D). Finally, we explored whether Usp9x influences Sufu ubiquitination modifications. When Usp9x was downregulated, there was a notable increase in the ubiquitination levels of Flag‐Sufu (Figure 3G). This result was further confirmed in the assessment of endogenous Sufu ubiquitination levels (Figure 3H). Moreover, overexpression of Usp9x, but not the C1566A mutant, was capable of reducing Sufu ubiquitination (Figure 3I). These findings collectively demonstrate that Usp9x acts as a deubiquitinase that stabilises Sufu by removing ubiquitin molecules from it. In addition, we assessed whether USP9X modulates SUFU ubiquitination in human MB cells. USP9X downregulation in DAOY cells (by knockdown or knockout) and in ONS‐76 cells (by knockdown) enhanced SUFU ubiquitination, which correlated with decreased SUFU protein stability (Figures S5A‒K, S6A‒C and S7A‒E).

### 3.4 USP9X inhibits SHH signalling through deubiquitinating SUFU

**FIGURE 4:**
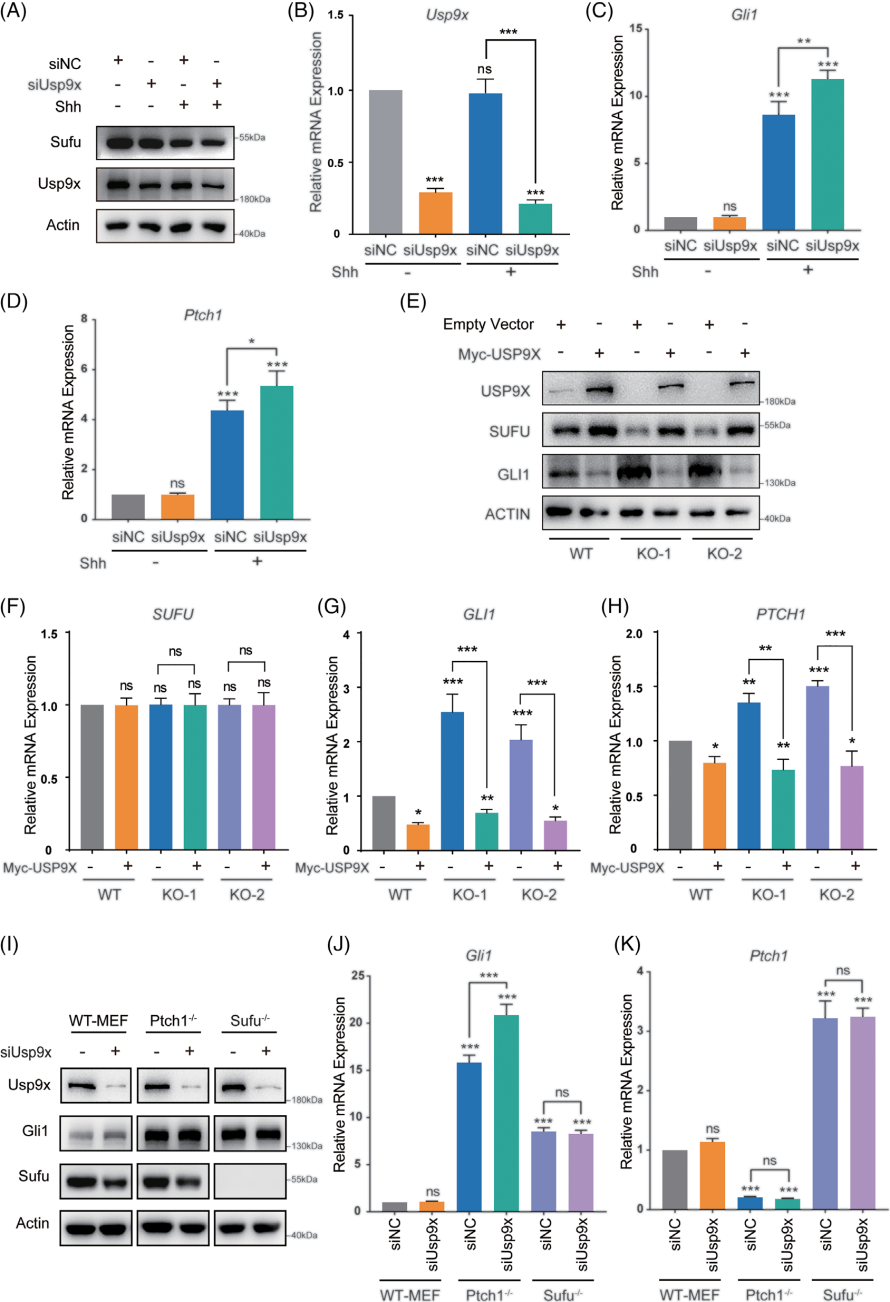
Usp9x suppresses Shh signalling through Sufu. (A) NIH3T3 cells transfected with the siNC or siUsp9x were treated with or without Shh ligand, and then Sufu and Usp9x protein levels were analysed by western blotting. The mRNA levels of *Usp9x* (B) and the Shh signalling pathway target genes *Gli1* (C) and *Ptch1* (D) were assessed by real‐time PCR. (E) Western analysis evaluating SUFU and GLI1 protein levels in *USP9X* knockout cells and rescue RK1M‐USP9X cells. The mRNA levels of *SUFU* (F), *GLI1* (G) and *PTCH1* (H) were analysed by real‐time PCR. (I) wild type mouse embryonic fibroblast (WT‒MEF), *Ptch1^−/−^* and *Sufu^−/−^* cells transfected with or without siUsp9x were analysed by Western blotting. The mRNA levels of *Gli1* (J) and *Ptch1* (K) in WT‒MEF, *Ptch1^−/−^* and *Sufu^−/−^* cells were assessed by real‐time PCR. ^*^ *p* < .05; ^**^ *p* < .01; ^***^ *p* < .001; ns, not significant.

Sufu, as a crucial negative regulator, is known to suppress Shh pathway activation mediated by the transcription factor Gli. Previous findings have indicated that Usp9x stabilises the protein levels of Sufu by deubiquitination, suggesting a potential role for Usp9x in modulating Shh signalling. Consequently, *Usp9x* siRNA was used in NIH3T3 cells to assess the transcription of Shh target genes. The results revealed that with a concomitant decrease in Sufu protein levels, Shh target genes *Gli1* and *Ptch1* were further upregulated in the Shh ligand and *Usp9x* siRNA treatment group (Figure 4A‒D), indicating that Usp9x acts as a negative regulator of Shh signalling. Consistently, in *USP9X* knockout cells, SUFU protein levels were markedly reduced, accompanied by upregulation of downstream Shh targets (Figure 4E‒H). Upon reintroduction of USP9X into the knockout cells, SUFU levels were restored, and the expression of SHH target genes, including Gli1 and Ptch1, was significantly attenuated (Figure 4E‒H).

To determine whether this negative regulation depends on Sufu, we downregulated Usp9x in wild type mouse embryonic fibroblast (WT‒MEF), *Ptch1^−/−^* and *Sufu^−/−^* cells and assessed the expression of Shh target genes. The results demonstrated that in WT‒MEF and *Ptch1^−/−^* cells, Usp9x knockdown decreased Sufu levels, demonstrating its ability to modulate Sufu protein levels (Figure 4I). Simultaneously, downregulating Usp9x in *Ptch1^−/−^* cells resulted in further upregulation of the target gene Gli1 transcription levels, whereas in *Sufu^−/−^* cells, the transcription levels of Gli1 and Ptch1 remained unaltered (Figure 4J,K). These findings collectively suggest that USP9X negatively regulates the SHH signalling pathway through the deubiquitination of SUFU.

### 3.5 SHH promotes USP9X degradation through the CMA pathway

**FIGURE 5:**
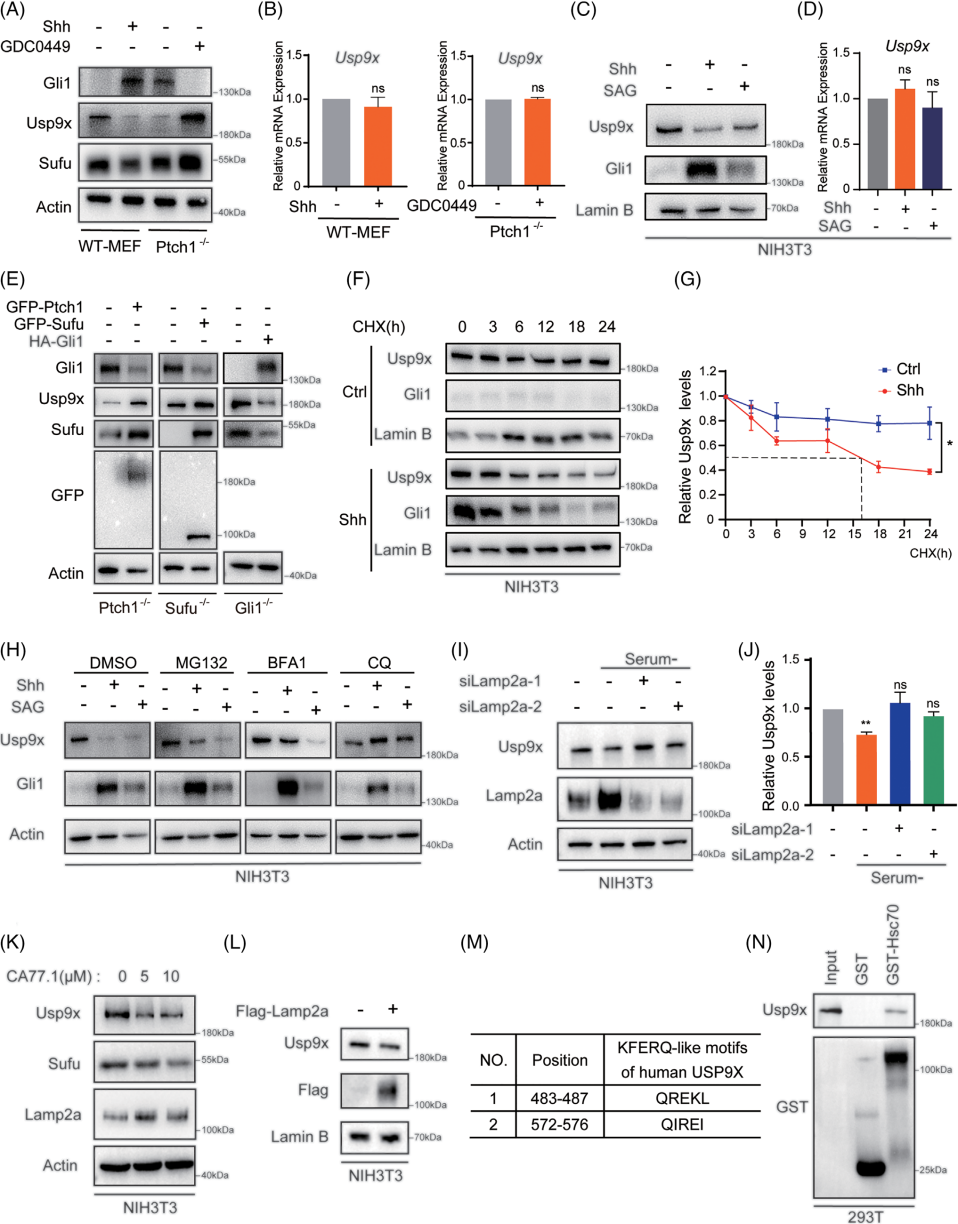
Shh promotes Usp9x degradation via chaperone‐mediated autophagy (CMA). (A) Western blot was conducted to evaluate the protein levels of Gli1, Usp9x and Sufu following treatment of wild type mouse embryonic fibroblast (WT‒MEF) cells with Shh ligand or *Ptch1^−/−^* cells with the inhibitor GDC‐0449. (B) The mRNA levels of *Usp9x* were assessed by Real‐time PCR in WT‒MEF and *Ptch1^−/−^* cells. (C) NIH3T3 cells were treated with either the Shh ligand or the Smoothened (SMO) agonist smoothened agonist (SAG), followed by Western blot analysis to examine Usp9x and Gli1 protein levels. (D) The mRNA levels of *Usp9x* were assessed by real‐time PCR. (E) *Ptch1^−/−^*, *Sufu^−/−^* and *Gli1^−/−^* cells were transfected with GFP‐Ptch1, GFP‐Sufu and HA‐Gli1 respectively, and cell lysates were subjected to immunoblotting analysis using indicated antibodies. Western analysis (F) and quantification thereof (G) of Usp9x protein levels in NIH3T3 cells with or without Shh ligand are shown. (H) NIH3T3 cells were treated with Shh ligand or SAG in the presence of the proteasome inhibitor MG132 or the lysosomal inhibitor chloroquine (CQ) or macroautophagy‐specific inhibitor BFA1, and then Usp9x and Gli1 protein levels were analysed. (I and J) immunoblot analyses with quantification of protein levels of Usp9x in NIH3T3 cells that were transfected with control siRNA or *Lamp2A* siRNA and then cultured with or without serum for 48 h. (K) NIH3T3 cells were treated with the CMA activator CA77.1, followed by Western blot analysis of Usp9x, Sufu and Lamp2A protein levels. (L) Immunoblot analysis of Usp9x protein levels in NIH3T3 cells following transfection with Flag‐Lamp2A. (M) KFERQ‐like motifs within the USP9X protein sequence were identified using KFERQ Finder v0.8. (N) Lysates from HEK293T cells expressing Myc‐USP9X were prepared and incubated with either glutathione S‐transferase (GST) or GST‐HSC70. GST pull‐down assays were subsequently conducted, and the bound proteins were analysed by Western blotting using Usp9x or GST antibodies. ^*^ *p* < .05; ^**^ *p* < .01; ns, not significant.

Our prior investigations uncovered an unexpected observation wherein treatment with the Shh ligand resulted in a marked reduction in Usp9x protein levels in NIH3T3 cells (Figure 4A, lanes 1 and 3), despite no detectable changes in RNA expression levels (Figure 4B). To corroborate this finding, we examined the effects of Shh pathway activation in both WT‒MEF and NIH3T3 cells. Stimulation with either Shh ligand or the agonist smoothened agonist (SAG) led to reduced protein levels of Usp9x and Sufu, whereas their transcription remained unchanged (Figure 5A‒D). Similarly, in *Ptch1^−/−^* cells, which exhibit constitutive Shh pathway activation, using Shh antagonist GDC‐0449 suppressed pathway activity, thereby restoring Usp9x and Sufu protein levels (Figure 5A,B). These findings indicate that the Shh signalling cascade regulates Sufu protein levels, in part, through the modulation of Usp9x. To delineate the molecular mechanisms underlying this regulation, we performed rescue experiments using *Ptch1^−/−^*, *Sufu^−/−^* and *Gli1^−/−^* cells, targeting key nodes of the Shh signalling pathway. Reintroduction of either Ptch1 or Sufu suppressed aberrant pathway activation, as evidenced by reduced Gli1 protein levels, and concomitantly restored Usp9x and Sufu protein levels. In contrast, reintroduction of the transcriptional activator Gli1 resulted in diminished Usp9x and Sufu protein levels, thereby positioning Usp9x regulation downstream of Gli1 (Figure 5E). These results collectively suggest that Shh signalling orchestrates the post‐transcriptional downregulation of Usp9x via Gli1‐dependent mechanisms, contributing to the modulation of Sufu stability within the pathway.

Further protein stability assays in NIH3T3 cells revealed that Shh ligand treatment markedly accelerated Usp9x degradation compared with controls, suggesting that Shh signalling promotes the degradation of Usp9x (Figure 5F,G). As eukaryotic protein degradation typically occurs via the proteasome or lysosome pathways, we tested the effects of inhibitors. Treatment with chloroquine, a lysosomal inhibitor, fully blocked Shh‐induced Usp9x downregulation, whereas the proteasome inhibitor MG132 had only minor effects, suggesting that Shh likely mediates the degradation of Usp9x via lysosome (Figure 5H). Lysosomal degradation mechanisms primarily involve macroautophagy and CMA.^32^ Notably, Shh‐mediated downregulation of Usp9x remained unaffected by the macroautophagy‐specific inhibitor Bafilomycin A1 (BFA1) (Figure 5H), indicating that CMA may be the predominant pathway for Usp9x degradation.

CMA selectively targets proteins containing KFERQ‐like motifs, recognised by the 70‐kDa heat shock cognate protein (Hsc70),^32^ and facilitates their lysosomal import via lysosome‐associated membrane protein type 2A (Lamp2A), the rate‐limiting component of the CMA translocation machinery.^33^ CMA activity is markedly induced under cellular stress conditions, such as nutrient deprivation.^32^ Upon serum withdrawal, NIH3T3 cells exhibited upregulation of Lamp2A protein levels accompanied by a concomitant reduction in Usp9x expression. In contrast, Lamp2A knockdown via siRNA abrogated the starvation‐induced decrease in Usp9x levels (Figure 5I–J). Supporting a role for CMA in Usp9x turnover, pharmacological activation of CMA with CA77.1 or overexpression of Flag‐tagged Lamp2A similarly resulted in reduced Usp9x protein abundance (Figure 5K,L). In analysis of the human USP9X amino acid sequence identified two canonical KFERQ‐like motifs (Figure 5M), consistent with its designation as a potential CMA substrate. Furthermore, GST pull‐down assays revealed a direct interaction between Usp9x and Hsc70 (Figure 5N), reinforcing the mechanistic link between CMA and Usp9x degradation. Collectively, these findings indicate that SHH signalling promotes USP9X degradation via the CMA pathway.

### 3.6 CMA inhibition markedly suppresses MB cell proliferation

**FIGURE 6:**
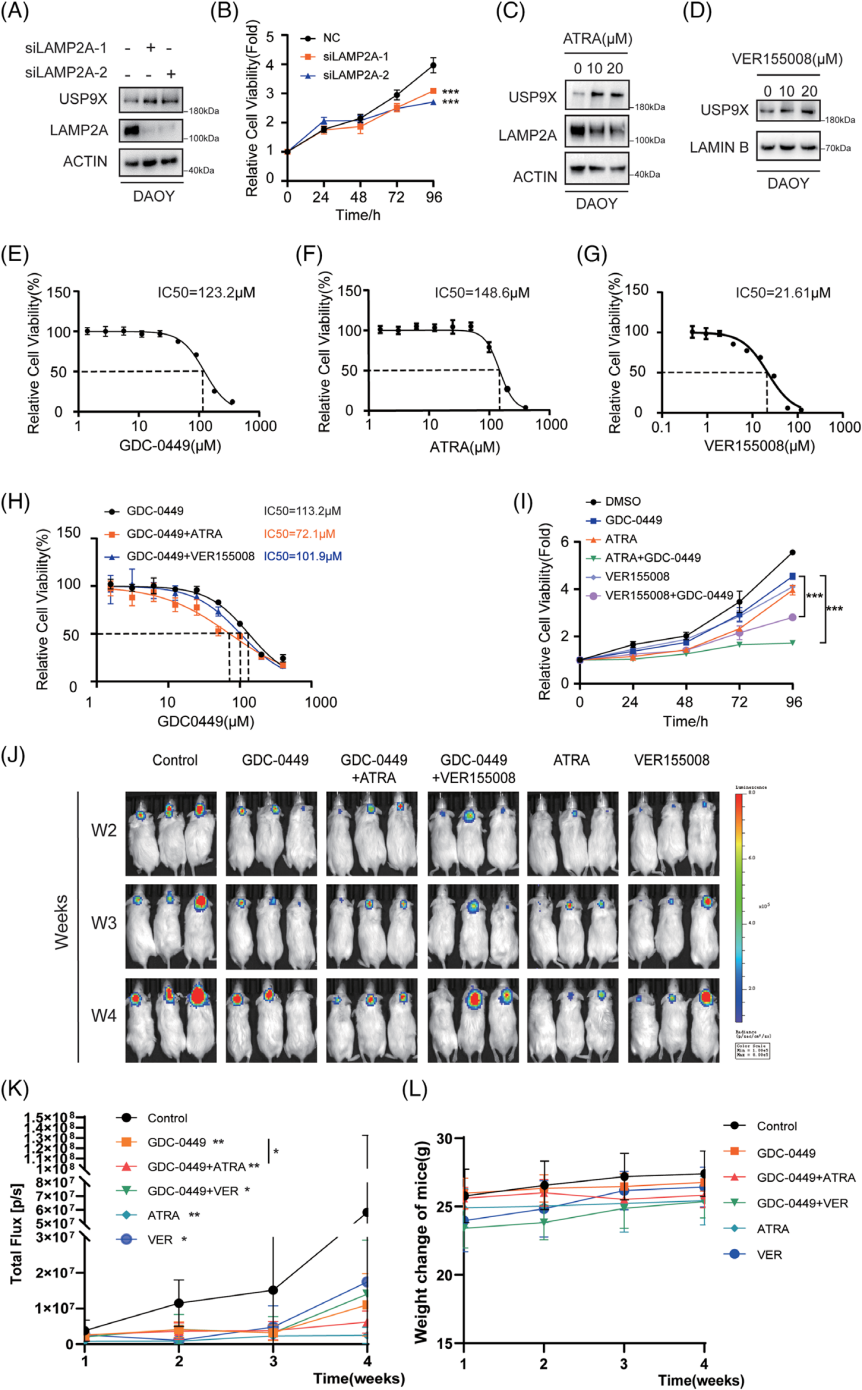
Synergistic suppression of medulloblastoma progression by the SHH pathway inhibitor GDC‐0449 in combination with all‐trans retinoic acid (ATRA) and VER155008. (A) DAOY cells transfected with the siNC or siUSP9X were analysed by Western blotting using indicated antibodies. (B) Cell counting kit‑8 (CCK‐8) assay were used to detect the effect of LAMP2A downregulation on DAOY cell viability. (C) Western blot analysis was performed to evaluate the protein levels of USP9X and LAMP2A following ATRA treatment in DAOY cells. (D) Western blot analysis was used to determine USP9X protein levels after VER155008 treatment in DAOY cells. The IC50 values for GDC‐0449 (E), ATRA (F) and VER155008 (G) against DAOY cells were provided. (H) The sensitivity of DAOY cells to GDC‐0449 was enhanced by the chaperone‐mediated autophagy (CMA) inhibitors ATRA and VER155008. (I) The proliferation of DAOY cells treated with GDC‐0449, ATRA or VER155008 was assessed using the CCK‐8 assay. (J) Representative in vivo imaging system (IVIS) bioluminescence images of mice bearing intracranial DAOY tumours treated with GDC‐0449, ATRA, VER155008, GDC‐0449 plus ATRA or GDC‐0449 plus VER155008 over a 4‐week period. (K) Quantification of tumour growth based on total photon flux within the region of interest measured weekly using Living Image software. (L) Body weight curves of mice in each treatment group during the 4‐week experimental period. ^*^ *p* < .05; ^**^ *p* < .01; ^***^ *p* < .001.

To determine whether CMA‐mediated degradation of USP9X occurs in MB, LAMP2A was silenced in SHH‐subtype DAOY cells. Western blot analysis revealed that LAMP2A knockdown markedly increased USP9X protein levels (Figure 6A). Given the well‐established role of USP9X in modulating MB cell proliferation, we further evaluated the impact of CMA inhibition on DAOY cell growth. CCK‐8 assay results demonstrated a significant reduction in proliferative capacity following LAMP2A silencing relative to control cells (Figure 6B). Consistently, pharmacological inhibition of CMA using ATRA or VER155008 led to a notable elevation in USP9X levels (Figure 6C,D). Given the critical role of CMA in regulating USP9X, we next investigated whether CMA inhibitors could suppress MB cell proliferation. A combinatorial therapeutic strategy was employed, integrating CMA inhibitors with the SHH pathway inhibitor GDC‐0449. The half‐maximal inhibitory concentrations (IC50) of GDC‐0449, ATRA and VER155008 were initially determined. As depicted in Figure 6E–H, monotherapy with each inhibitor resulted in relatively higher IC50 values. However, the co‐administration of either ATRA or VER155008 with GDC‐0449 significantly reduced the IC50, indicating enhanced efficacy. Moreover, dual treatment with GDC‐0449 and either CMA inhibitor elicited a more pronounced suppressive effect on MB cell growth (Figure 6I). To further evaluate the therapeutic effect of the combination treatment with ATRA, VER155008 and GDC‐0449 in vivo, luciferase‐expressing DAOY cells were implanted into the cerebellum of nude mice and randomised into six treatment groups. Consistent with the in vitro results in Figure 6I, single‐agent treatments reduced tumour growth relative to control. Importantly, the combination of GDC‐0449 and ATRA showed stronger tumour suppression than GDC‐0449 alone, whereas VER155008 combined with GDC‐0449 did not result in a notable additional effect (Figure 6J‒L). In summary, these findings highlight the potent antitumour effects of combinatorial treatment with GDC‐0449 and either ATRA or VER155008, underscoring their potential clinical utility in MB therapy.

## 4 DISCUSSION

As one of the most widespread post‐translational modifications, ubiquitination—and its reversal by DUB enzymes—constitutes a dynamic and reversible mechanism governing protein stability and functional activity.^22^ Dysregulation of this system is a key driver of tumourigenesis. Modulating protein ubiquitination and deubiquitination has emerged as a promising strategy in anticancer drug development.^34^, ^35^ Within the SHH signalling pathway—critical for embryonic development and aberrantly activated in various cancers, including SHH MB, core components such as PTCH1, SMO and GLI family transcription factors are tightly regulated by the ubiquitin–proteasome system (UPS).^36^ These components are modulated by various E3 ligases and DUBs, underscoring the importance of ubiquitin‐mediated proteostasis in SHH pathway regulation.^36^ However, the role of deubiquitination in modulating SUFU, a key negative regulator of SHH signalling, remains poorly understood. Here, we identify USP9X stabilises SUFU and suppresses SHH signalling, thereby restraining MB progression. This mechanism adds an additional regulatory layer to the current model of SUFU control, highlighting that SUFU abundance is not solely determined by ubiquitin ligase activity but is also actively safeguarded by deubiquitinases in a context‐dependent manner.

USP9X, a member of the USP family, governs the stability of key regulatory proteins, thereby exerting context‐dependent effects on tumour progression.^28^ In glioblastoma, USP9X stabilises ALDH1A3 to sustain cancer stem cell properties,^29^ while it enhances HIF‐1α stability, promoting stemness under hypoxia in triple‐negative breast cancer.^37^ Conversely, USP9X functions as a tumour suppressor in colorectal cancer by stabilising FBW7 and preventing c‐Myc accumulation,^38^ and in pancreatic cancer, it deubiquitinates LATS2, thereby restraining YAP/TAZ‐driven oncogenesis.^39^, ^40^ These observations underscore the multifaceted roles of USP9X in tumour biology. However, the mechanisms governing its regulation remain incompletely defined. In this study, we identify the SHH pathway as a previously unrecognised modulator of USP9X stability, demonstrating that SHH signalling facilitates USP9X degradation via CMA.

CMA is a selective lysosomal degradation pathway in which proteins bearing KFERQ‐like motifs are recognised by HSC70 and translocated into lysosomes via LAMP2A for proteolysis.^33^, ^41^ Although sustained activation of CMA has been implicated in several cancers—including lung, colorectal, gastric and liver malignancies—its functional role in SHH‐MB remains undefined.^42^ We show that USP9X is targeted by CMA, and its degradation leads to the destabilisation of SUFU, a key negative regulator of SHH signalling. This destabilisation results in enhanced GLI activity and subsequent tumour proliferation, highlighting a novel mechanism by which SHH signalling influences MB progression. This finding expands the regulatory landscape of SHH signalling beyond the UPS and positions CMA as an integral modulator of pathway output. Importantly, our findings demonstrate that pharmacological inhibition of CMA restores USP9X protein levels, resulting in SUFU stabilisation. The combination of CMA inhibitors—specifically ATRA or VER155008—with the SMO inhibitor GDC‐0449 produces a synergistic antiproliferative effect in SHH‐MB cells, suggesting that dual targeting of CMA and SHH signalling could overcome intrinsic or acquired resistance to SMO monotherapy.^43^, ^44^ This is particularly relevant given the clinical limitations of GDC‐0449, which, despite being FDA‐approved for SHH‐driven tumours, often exhibits diminished efficacy due to resistance mechanisms within the SHH pathway.^45^, ^46^ Of note, ATRA is already widely used in the clinical management of acute promyelocytic leukaemia, where it promotes cellular differentiation and inhibits proliferation.^47^ Our findings suggest that ATRA, through its capacity to inhibit CMA, may be repurposed to enhance SHH pathway inhibition in MB. However, given that CMA regulates numerous cellular proteins, systemic inhibition may lead to significant off‐target effects and toxicity, limiting its clinical use. Alternatively, deubiquitinase‐targeting chimeras (DUBTACs),^48^ recently developed heterobifunctional small molecules that link a deubiquitinase recruiter to a protein‐targeting ligand, offer a promising strategy to selectively stabilise SUFU. This approach could mitigate the limitations of CMA and USP9X inhibition and provide a novel therapeutic avenue for MB.

**FIGURE 7:**
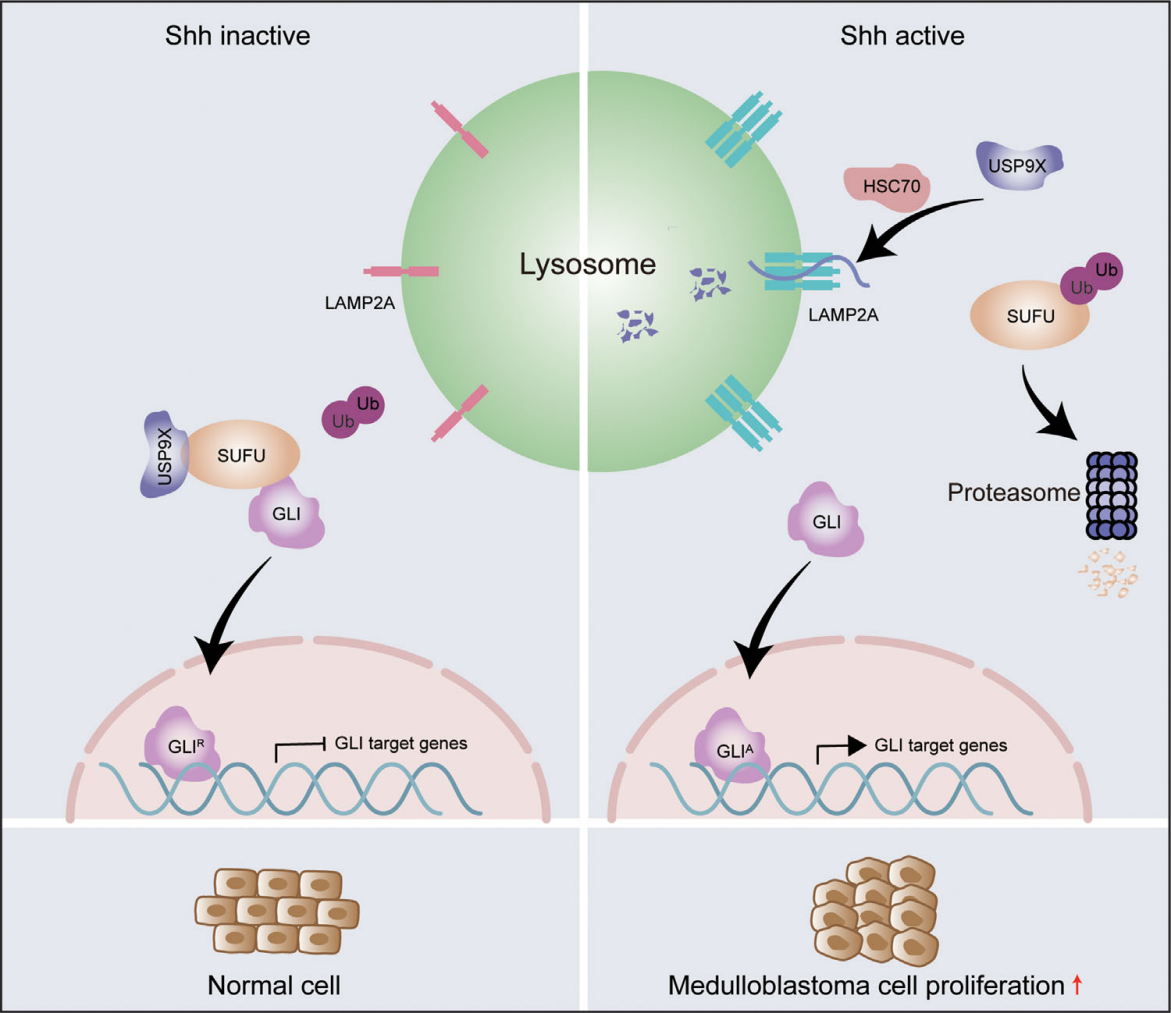
Model of suppressor of fused (SUFU) regulation by the sonic Hedgehog (SHH)‒ubiquitin‐specific protease 9X (USP9X) axis via chaperone‐mediated autophagy (CMA) and the ubiquitin–proteasome pathway in sonic Hedgehog molecular subtype Medulloblastoma (SHH‐MB) progression.

In summary, our findings uncover a previously unrecognised mechanism whereby SHH and CMA jointly regulate MB progression (Figure 7) and highlight the CMA–USP9X axis as a potential therapeutic target. These results not only advance our understanding of SHH‐MB pathogenesis but also propose a framework to overcome limitations of current targeted therapies.

## AUTHOR CONTRIBUTIONS

*Writing—original draft, methodology, formal analysis, data curation and conceptualisation*: Binbin Gao and Qin Zhu. *Methodology, formal analysis and data curation*: Lun Kuang. *Writing—original draft, methodology, formal analysis and data curation*: Jiahui Li. *Methodology*: Qingyue Meng, Bo'ang Han, Yu Wang and Xiangxiang Zhang. *Validation and methodology*: Xinyi Zhang. *Supervision, conceptualisation and resources*: Xinfa Wang. *Supervision and conceptualisation*: Tingting Yu. *Funding acquisition, writing—review and editing, supervision, project administration and conceptualisation*: Shen Yue. *Funding acquisition, writing—review and editing, supervision, project administration and conceptualisation*: Chen Liu.

## CONFLICT OF INTEREST STATEMENT

The authors declare they have no conflicts of interest. Generative AI and AI‐assisted technologies were not used in the preparation of this work.

## ETHICS STATEMENT

All animal procedures were conducted in accordance with institutional guidelines and were authorised by the Animal Core Facility of Nanjing Medical University (approval number: 14030113‐4). For research involving human subjects, informed consent was obtained from all participants, either verbally or in written form, prior to sample collection.

## Supporting information



Supporting information
