# Supplementary material for: CMA‐mediated USP9X degradation promotes SHH medulloblastoma progression by facilitating SUFU ubiquitination
Source: Clin Transl Med. 2026 Mar 8;16(3):e70635. doi: 10.1002/ctm2.70635 (PMC12968324; doi:10.1002/ctm2.70635)
Supplement: Supplementary file 1 — Supporting information [file CTM2-16-e70635-s001.docx]

**Supplementary Information**

**Supplementary Figures**

**
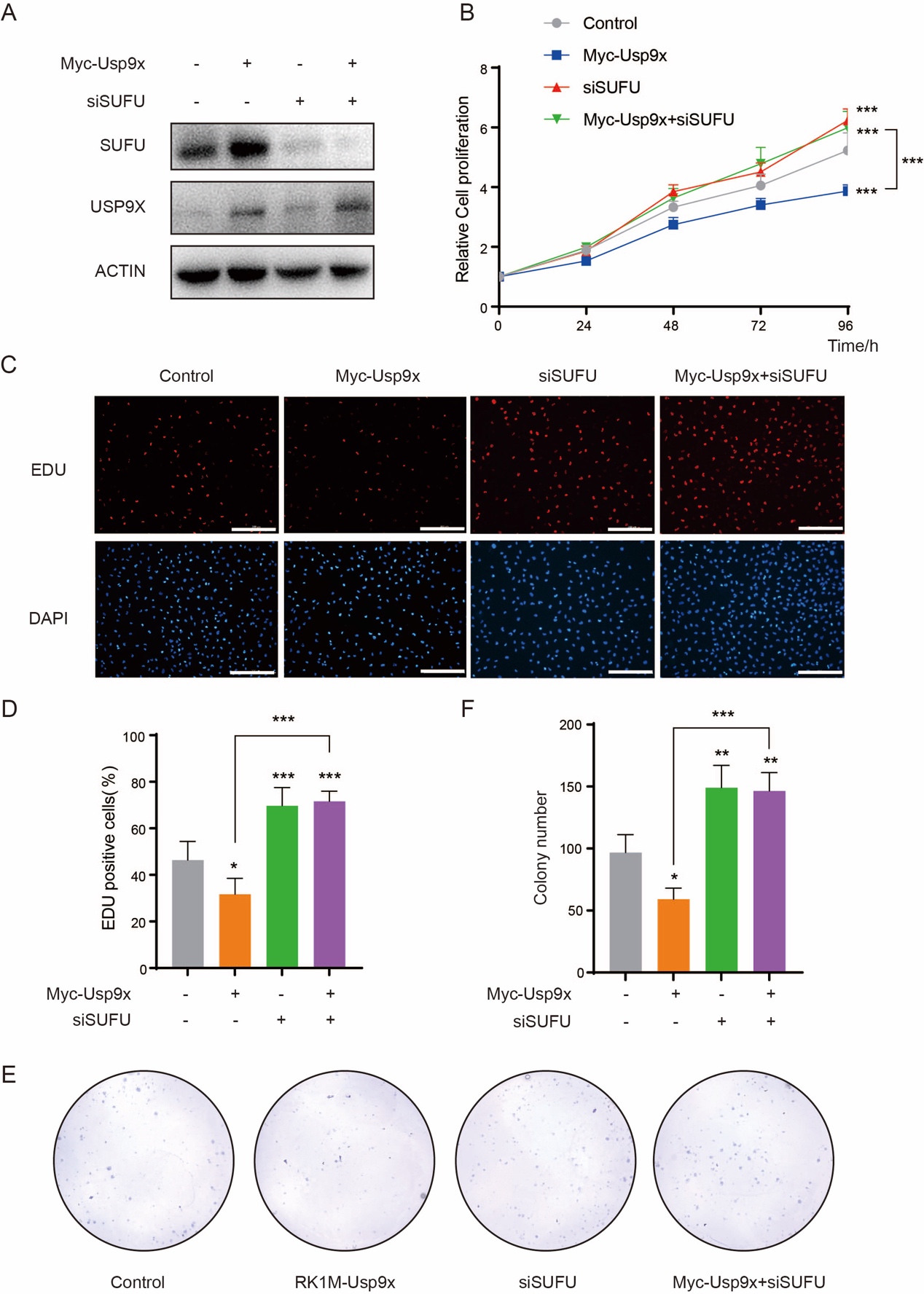
**

**sFigure 1. USP9X inhibits DAOY cell proliferation via SUFU. (A)** Western blot analysis verifying the knockdown efficiency of SUFU and the overexpression of USP9X in DAOY cells. Cell counting kit‑8(CCK8) **(B)**, EDU**(C-D)** and colony formation**(E-F)** assay were used to detect the effects of USP9X overexpression, SUFU knockdown, and their combination on DAOY cell viability. ∗P < 0.05; ∗∗P < 0.01; ∗∗∗P < 0.001.


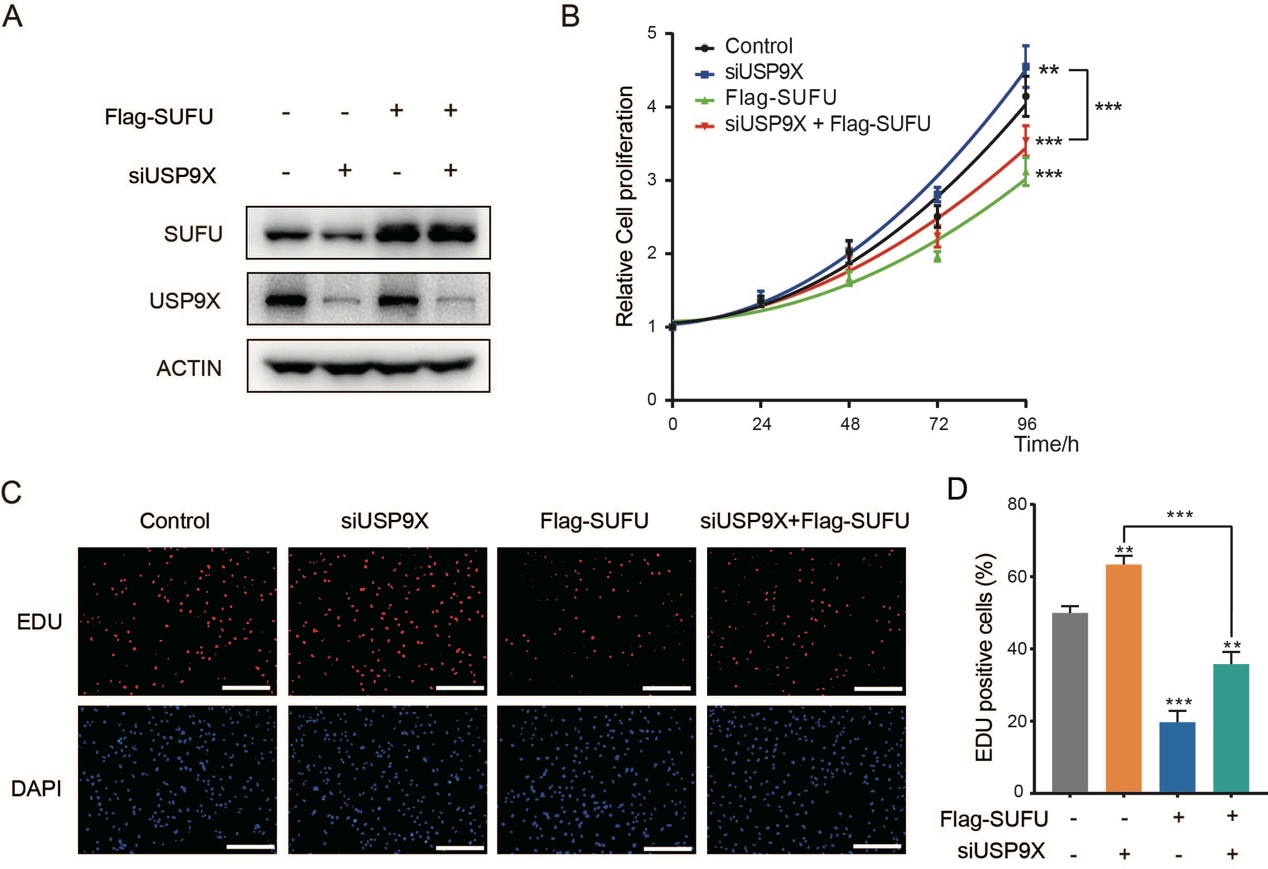


**sFigure 2. USP9X suppresses DAOY cell proliferation through SUFU-mediated regulation. (A)** Western blot analysis verifying the knockdown efficiency of USP9X and the overexpression of SUFU in DAOY cells. Cell counting kit‑8(CCK8) **(B)**, EDU**(C)** assay were used to detect the effects of SUFU overexpression, USP9X knockdown, and their combination on DAOY cell viability. **(D)** Quantitative data from three independent experiments performed as in **(C)**. ∗∗P < 0.01; ∗∗∗P < 0.001.


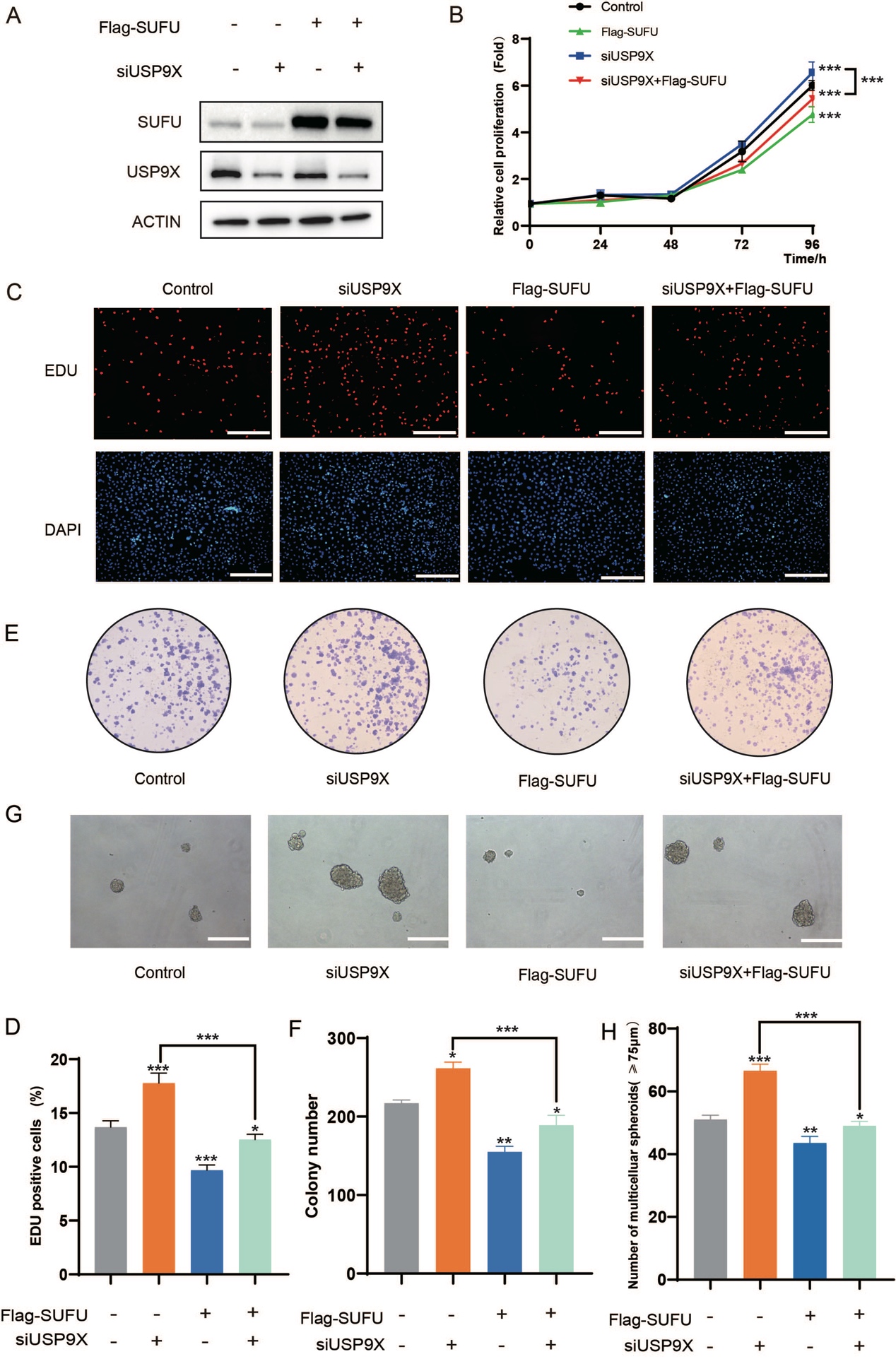


**sFigure 3. USP9X suppresses ONS-76 cell proliferation through SUFU-mediated regulation. (A)** Western blot analysis verifying the knockdown efficiency of USP9X and the overexpression of SUFU in ONS-76 cells. Cell counting kit‑8(CCK8) **(B)**, EDU**(C-D)** and colony formation**(E-F)** assay were used to detect the effects of SUFU overexpression, USP9X knockdown, and their combination on ONS-76 cell viability. **(G-H)** Sphere formation assay to detect the stemness of ONS-76 cells and statistical results.∗P < 0.05; ∗∗P < 0.01; ∗∗∗P < 0.001.


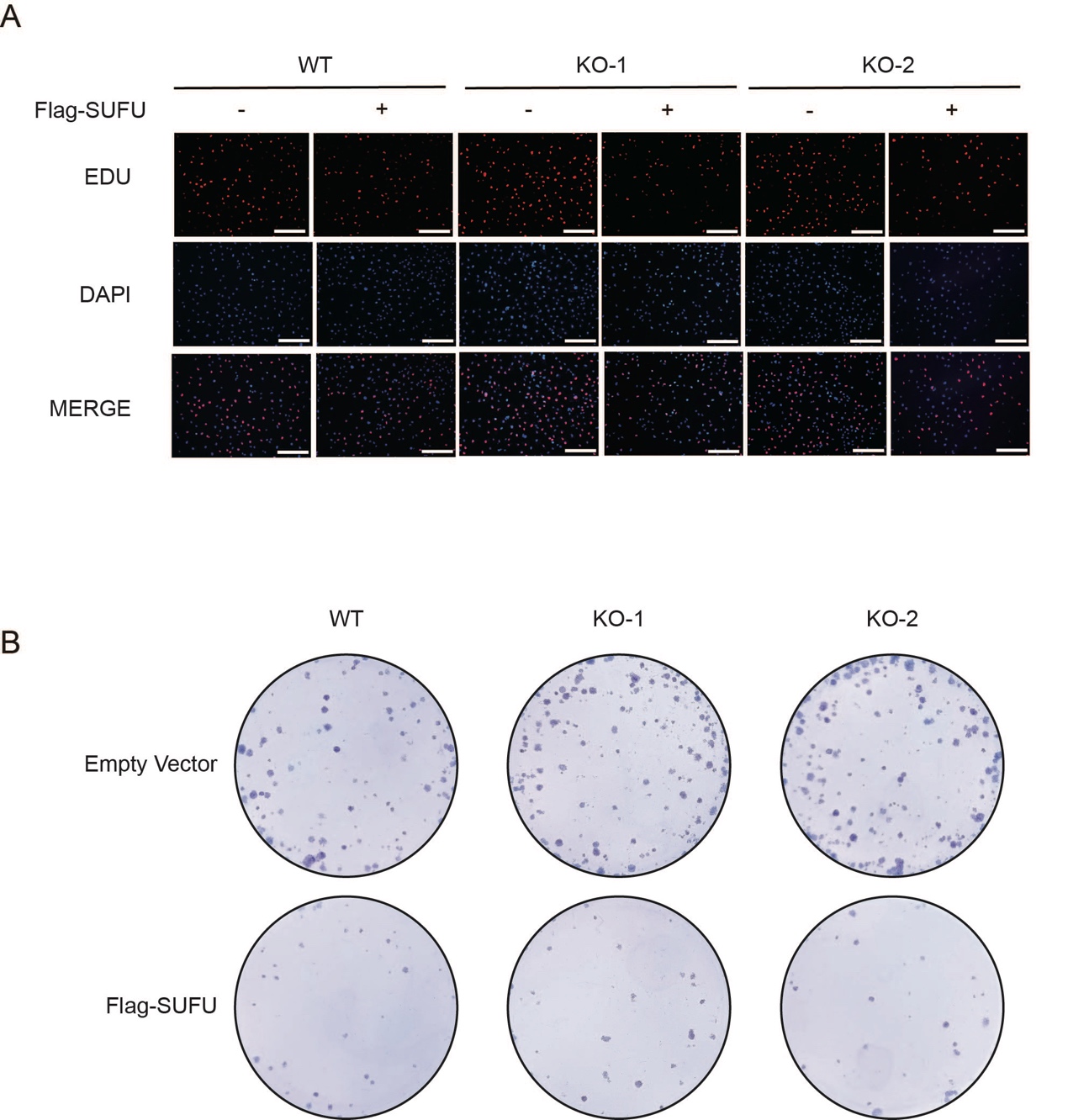


**sFigure 4. USP9X modulates DAOY cell proliferation through SUFU regulation.** EDU**(A)** and colony formation**(B)** assay were used to detect the effect of Flag-SUFU overexpression on DAOY cell viability in *USP9X* knockout cells.


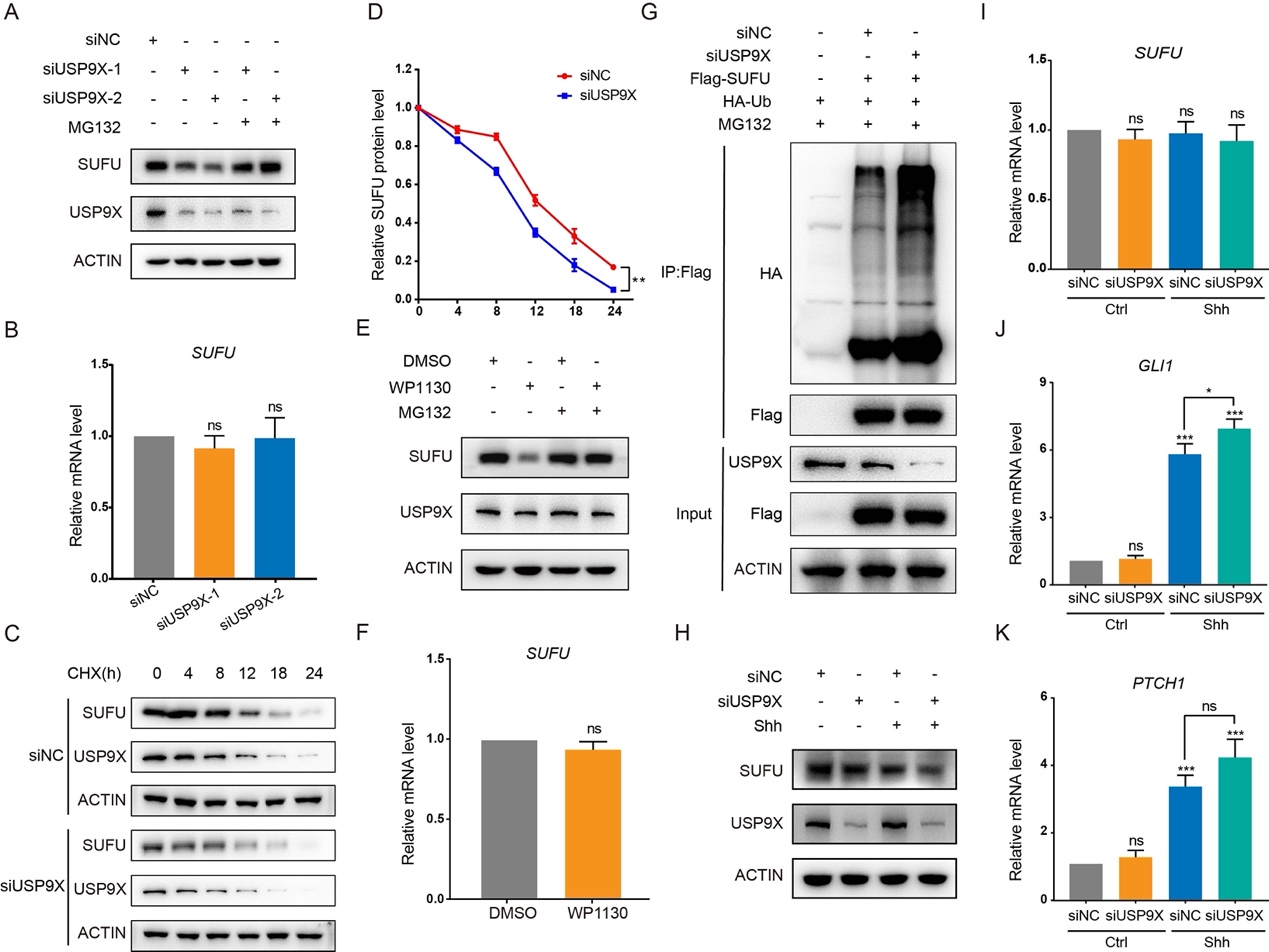


**sFigure 5. USP9X deubiquitinates SUFU in DAOY cells. (A)** DAOY cells transfected with the indicated siRNA were treated with or without MG132, and then SUFU and USP9X were analyzed. **(B)** DAOY cells expressing siNC, siUSP9X-1, siUSP9X-2 were analyzed by Real-time PCR. Western analysis **(C)** and quantification thereof **(D)** of SUFU protein levels in DAOY cells transfected with siUSP9X are shown. Protein synthesis was blocked with CHX treatment. **(E-F)** DAOY cells were treated with or without WP1130 in the absence or presence of MG132, and cell lysates were subjected to immunoblotting analysis using indicated antibodies. **(G)** DAOY cells were cotransfected with indicated siRNA, Flag-SUFU, and HA-Ub in the presence of MG132, and cell lysates were subjected to IP with Flag beads followed by immunoblotting analysis. **(H)** DAOY cells transfected with the siNC or siUSP9X were treated with or without Shh ligand, and then SUFU and USP9X protein levels were analyzed by western blotting. The mRNA levels of *SUFU* **(I)** and the Shh signaling pathway target genes *GLI1* **(J)** and *PTCH1* **(K)** were assessed by Real-time PCR. ∗P < 0.05; ∗∗P < 0.01; ∗∗∗P < 0.001; ns, not significant.


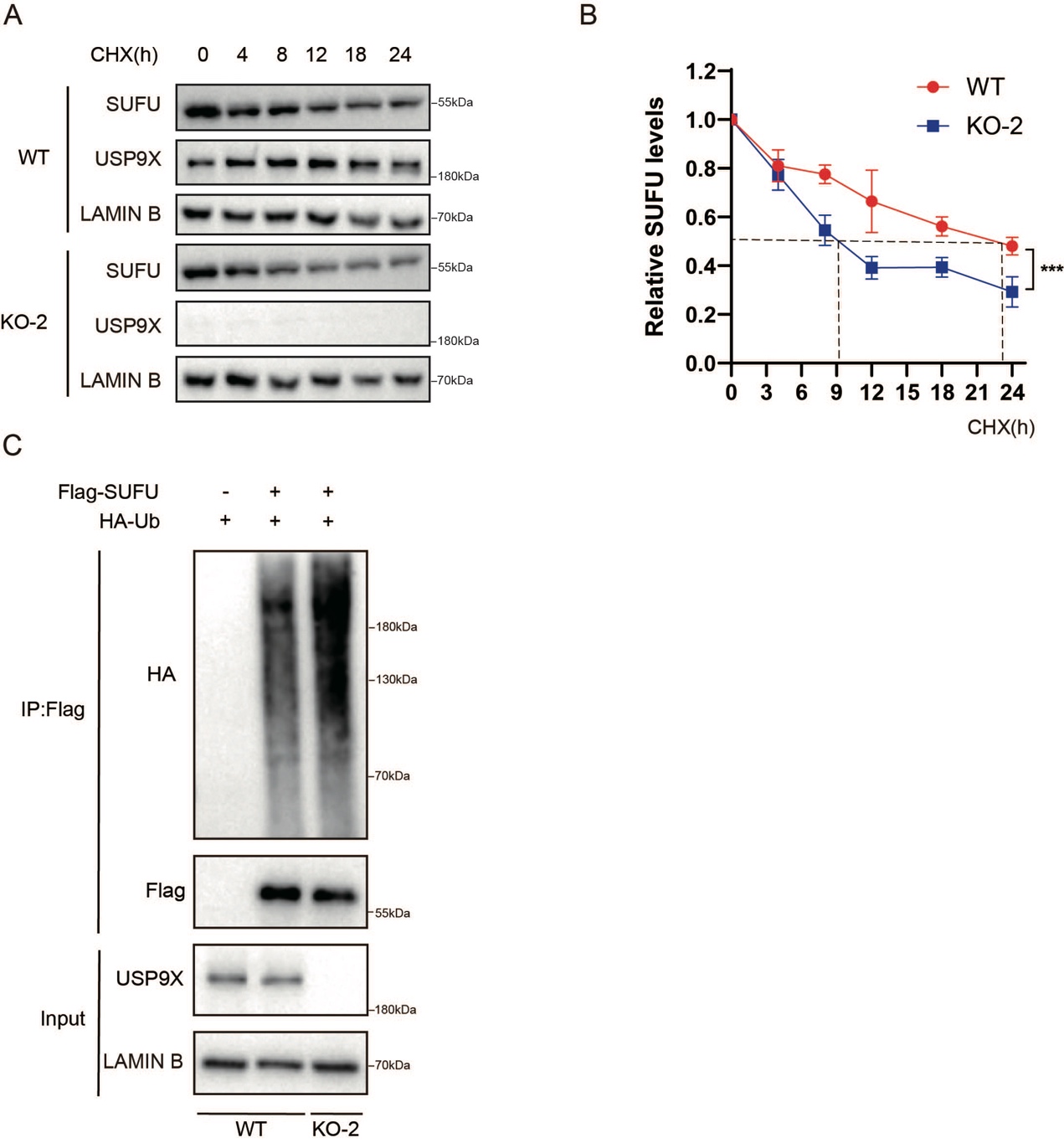


**sFigure 6. USP9X deubiquitinates SUFU in DAOY cells.** Western analysis **(A)** and quantification thereof **(B)** of SUFU protein levels in *USP9X* WT and knockout DAOY cells. Protein synthesis was blocked with CHX treatment. **(C)** *USP9X* WT and knockout DAOY cells were transfected with Flag-SUFU and HA-Ub. Cell lysates were subjected to IP with Flag beads, followed by immunoblotting analysis.


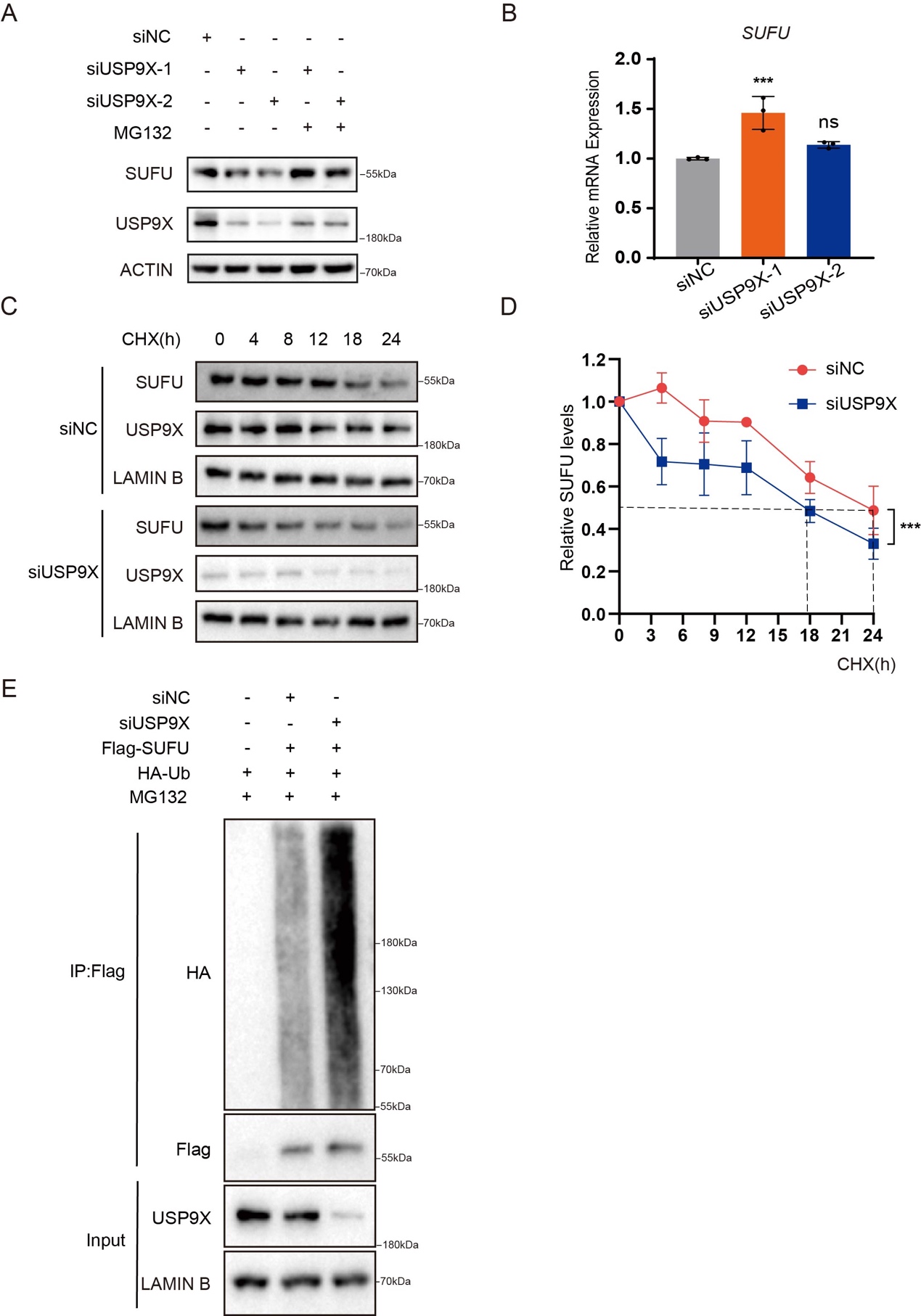


**sFigure 7. USP9X deubiquitinates SUFU in ONS-76 cells. (A)** ONS-76 cells transfected with the indicated siRNA were treated with or without MG132, and then SUFU and USP9X were analyzed. **(B)** ONS-76 cells expressing siNC, siUSP9X-1, siUSP9X-2 were analyzed by real-time PCR. Western analysis **(C)** and quantification thereof **(D)** of SUFU protein levels in ONS-76 cells transfected with siUSP9X are shown. Protein synthesis was blocked with CHX treatment. **(E)** ONS-76 cells were cotransfected with indicated siRNA, Flag-SUFU, and HA-Ub in the presence of MG132, and cell lysates were subjected to IP with Flag beads followed by immunoblotting analysis. ∗∗∗P < 0.001; ns, not significant.

**Supplementary Tables**

**sTable 1**

siRNA sequences

| Gene | Organisms | sense（5'-3'） | antisense（5'-3'） |
| --- | --- | --- | --- |
| ***USP9X-1*** | Homo | GCACUGAAUGAAGUUAAUATT | UAUUAACUUCAUUCAGUGCTT |
| ***USP9X-2*** | Homo | CCUGAUAGCUCUUCUGAUUTT | AAUCAGAAGAGCUAUCAGGTT |
| ***Usp9x-1*** | Mus | GCACUGAAUGAGGUUAAUATT | UAUUAACCUCAUUCAGUGCTT |
| ***Usp9x-2*** | Mus | GCAGGCAAGUAGAUGACUUTT | AAGUCAUCUACUUGCCUGCTT |
| ***SUFU*** | Homo | CCUCAUUCCUCUCUGCCUATT | UAGGCAGAGAGGAAUGAGGTT |
| ***LAMP2A-1*** | Homo | GCAGUGCAGAUGACGACAATT | UUGUCGUCAUCUGCACUGCTT |
| ***LAMP2A-2*** | Homo | GCCUUGGCAGGAGUACUUATT | UAAGUACUCCUGCCAAGGCTT |
| ***Lamp2a-1*** | Mus | GCCGUUCAGUCCAAUGCAUTT | AUGCAUUGGACUGAACGGCTT |
| ***Lamp2a-1*** | Mus | GGUCUCAAGCGCCAUCAUATT | UAUGAUGGCGCUUGAGACCTT |

**sTable 2**

Antibodies

| **Antibodies** | **Source** | **Identifier** | **Application** |
| --- | --- | --- | --- |
| Rabbit anti Gli1 | CST, USA | 2534 | WB(1:1000) |
| Rabbit anti HA | Proteintech, China | 51064-2-AP | WB(1:5000) |
| Rabbit anti Sufu | Proteintech, China | 26759 | WB(1:1000),IHC(1:100) |
| Rabbit anti Ki67 | Abcam, USA | ab15580 | IHC(1:200),IF(1:200) |
| Rabbit anti Usp9x | Proteintech, China | 55054 | WB(1:1000) |
| Rabbit anti Usp9x | HUABIO, China | ET7108-08 | IHC(1:200) |
| Mouse anti Usp9x | Santa Cruz, USA | sc-365353 | IF(1:200) |
| Rabbit anti GFP | Abcam, USA | ab290 | WB(1:1000) |
| Mouse anti Myc | Sigma, USA | M4439 | WB(1:5000) |
| Mouse anti Flag | Sigma, USA | F1804 | WB(1:2000) |
| Rabbit anti GST | HUABIO, China | ET1611-47 | WB(1:1000) |
| Rabbit anti LAMP2A | HUABIO, China | ET1601-24 | WB(1:5000) |
| Mouse anti β-actin | Santa Cruz, USA | SC4778 | WB(1:2000) |
| Mouse anti Lamin B1 | Proteintech, China | 66095-1-Ig | WB(1:2000) |
| Alexa Fluor 488 Mouse Secondary Antibody | Thermo Scientific, USA | A-21202 | IF(1:200) |
| Alexa Fluor 594 Rabbit Secondary Antibody | Thermo Scientific, USA | A-32754 | IF(1:200) |
| Goat anti-rabbit IgG HRP | Jackson Immuno Research, USA | 111-035-003 | WB(1:5000) |
| Goat anti-mouse IgG HRP | Jackson Immuno Research, USA | 115-035-003 | WB(1:5000) |

**sTable 3**

Quantitative Real-time PCR Primers

| Gene | Organisms | sense（5'-3'） | antisense（5'-3'） |
| --- | --- | --- | --- |
| Gli1 | Mus | CTCAAACTGCCCAGCTTAACCC | TGCGGCTGACTGTGTAAGCAGA |
| Ptch1 | Mus | TGCTGTGCCTGTGGTCATCCTGATT | CAGAGCGAGCATAGCCCTGTGGTTC |
| Sufu | Mus | CTCCAGGTTACCGCTATCGTC | CACTTGGTCCGCTGTTCCTG |
| Usp9x | Mus | AAACTGTGAGACTGGGAAGTAG | CGATCACAAAGATAAACCGCAT |
| Actin | Mus | CATTGCTGACAGGATGCAGAAGG | TGCTGGAAGGTGGACAGTGAGG |
| GLI1 | Homo | AGCTAGAGTCCAGAGGTTCAA | TAGACAGAGGTTGGGAGGTAAG |
| PTCH1 | Homo | GGGTGGCACAGTCAAGAACAG | TACCCCTTGAAGTGCTCGTACA |
| SUFU | Homo | ACATGCTGCTGACAGAGGAC | CACTGCTGGGCTGAGTGTAG |
| USP9X | Homo | GCCAGATGACCAAGATGCTCCAG | GGTTGTTCATGTGATGTGCTGCTG |
| ACTIN | Homo | CATCGAGCACGGCATCGTCA | TAGCACAGCCTGGATAGCAAC |

**sTable 4**

USP9X sgRNA oligos

| Name | Sequence (5'-3') |
| --- | --- |
| sgRNA-1 | GACGTGTACCATTGTAGATATGG |
| sgRNA-2 | GGACTCAATTGTTGTTCTGTAGG |

USP9X PCR oligos

| Name | Sequence (5'-3') |
| --- | --- |
| P1 | GCAGTGTTTTGATCTTGTAAATCGC |
| P2 | TTTCTGGCTGGATAGAAAAAGGGA |
